# Dosage compensation and sex-specific epigenetic landscape of the X chromosome in the pea aphid

**DOI:** 10.1186/s13072-017-0137-1

**Published:** 2017-06-15

**Authors:** Gautier Richard, Fabrice Legeai, Nathalie Prunier-Leterme, Anthony Bretaudeau, Denis Tagu, Julie Jaquiéry, Gaël Le Trionnaire

**Affiliations:** 1 EGI, UMR 1349, INRA, Institut de Génétique, Environnement et Protection des Plantes (IGEPP), Domaine de la Motte, BP 35327, Le Rheu, France; 2 BIPAA, UMR 1349, INRA, Institut de Génétique, Environnement et Protection des Plantes (IGEPP), Campus Beaulieu, Rennes, France; 3 Genscale, INRIA, IRISA, Campus Beaulieu, Rennes, France; 4 Genouest, INRIA, IRISA, Campus Beaulieu, Rennes, France; 5 CNRS, UMR 6553, EcoBio, University of Rennes 1, 35042 Rennes, France

**Keywords:** X chromosome, Dosage compensation, Transcriptomics, Open chromatin, Non-model organism, Pea aphid, *Acyrthosiphon pisum*, Formaldehyde-Assisted Isolation of Regulatory Elements (FAIRE)

## Abstract

**Background:**

Heterogametic species display a differential number of sex chromosomes resulting in imbalanced transcription levels for these chromosomes between males and females. To correct this disequilibrium, dosage compensation mechanisms involving gene expression and chromatin accessibility regulations have emerged throughout evolution. In insects, these mechanisms have been extensively characterized only in Drosophila but not in insects of agronomical importance. Aphids are indeed major pests of a wide range of crops. Their remarkable ability to switch from asexual to sexual reproduction during their life cycle largely explains the economic losses they can cause. As heterogametic insects, male aphids are X0, while females (asexual and sexual) are XX.

**Results:**

Here, we analyzed transcriptomic and open chromatin data obtained from whole male and female individuals to evaluate the putative existence of a dosage compensation mechanism involving differential chromatin accessibility of the pea aphid’s X chromosome. Transcriptomic analyses first showed X/AA and XX/AA expression ratios for expressed genes close to 1 in males and females, respectively, suggesting dosage compensation in the pea aphid. Analyses of open chromatin data obtained by Formaldehyde-Assisted Isolation of Regulatory Elements (FAIRE-seq) revealed a X chromosome chromatin accessibility globally and significantly higher in males than in females, while autosomes’ chromatin accessibility is similar between sexes. Moreover, chromatin environment of X-linked genes displaying similar expression levels in males and females—and thus likely to be compensated—is significantly more accessible in males.

**Conclusions:**

Our results suggest the existence of an underlying epigenetic mechanism enhancing the X chromosome chromatin accessibility in males to allow X-linked gene dose correction between sexes in the pea aphid, similar to Drosophila. Our study gives new evidence into the comprehension of dosage compensation in link with chromatin biology in insects and newly in a major crop pest, taking benefits from both transcriptomic and open chromatin data.

**Electronic supplementary material:**

The online version of this article (doi:10.1186/s13072-017-0137-1) contains supplementary material, which is available to authorized users.

## Background

**Fig. 1:**
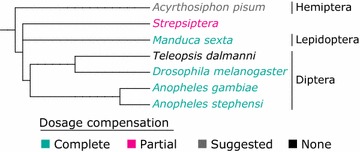
Phylogenetic tree of the main insect species for which dosage compensation has been studied. This tree has been generated using PhyloT (http://phylot.biobyte.de/) and the NCBI taxonomy. The *color* defines the dosage compensation type (complete dosage compensation in *green*, partial dosage compensation in *pink*, suggested dosage compensation in *gray*, and no dosage compensation in *black*)

The sex of individuals relies in many organisms upon morphologically differentiated sex chromosomes. Those chromosomes are referred to as X, Y, Z and W chromosomes depending on which sex is heterogametic. In organisms with male heterogamety, females encompass two X chromosomes, while males possess only one X, accompanied or not by a Y chromosome (XX/XY or XX/X0 systems) [1]. In these organisms showing a degenerated or an absence of Y chromosome, there are an imbalanced number of X-linked alleles between males and females that might induce differential transcription levels for those genes between sexes [2]. Such a disequilibrium needs to be corrected, especially for X-linked genes that interact with autosomal genes, since reduced gene dose in the heterogametic sex might have deleterious phenotypic consequences [2–5]. Dosage compensation mechanisms are thought to have evolved to correct such disequilibrium. These mechanisms tend to generate equilibrated X-linked and autosomal transcript levels, often resulting in XX/AA and X/AA expression ratios equal to 1 in both the homogametic (XX) and the heterogametic (XY or X0) sexes, and consequently of XX/X ratios also equal to 1, as described in several model organisms such as Eutherian mammals [2, 6–9], *Caenorhabditis elegans* [9–11] and the insect model *Drosophila melanogaster* [12–14]. Recently, complete dosage compensation in other male heterogametic insect species (Fig. 1)—namely *Anopheles stephensi* [15], *Anopheles gambiae* [16] and *Manduca sexta* [17]—has been demonstrated using transcriptomic data. A partial dosage compensation was found in *Strepsiptera* [18], and no compensation was detectable in *Teleopsis dalmanni* [19].

Various mechanisms have evolved to counteract the deleterious effects of different doses of X-linked genes in males and females. These mechanisms are all based on the modulation of chromatin accessibility of the X chromosome(s) in one sex in order to ensure that both sex’s somatic—and sometimes germ cells [20]—show similar transcription levels for X-linked genes [21]. In mammals, dosage compensation mechanisms take place in the female where one of the two X chromosomes—either the maternal or paternal one—is entirely inactivated, thus balancing the X/A transcription level in males and females. This transcriptional regulation is achieved by the progressive depletion of active marks such as H3K4me1 and H3K9ac [22] and the enrichment of histone macroH2A1 [23] on the female’s inactive X chromosome. Moreover, the histone repressive mark H3K27me3 is enriched on this inactive chromosome through the action of the X-inactive specific transcript (Xist), a long noncoding RNA (lncRNA) [24]. In *C. elegans* (a XX/X0 system), dosage compensation also takes place in the homogametic sex, where the two X chromosomes display halved transcription levels [25]. Reduced transcription is allowed by an enrichment of the repressive H4K20me1 histone mark and a depletion of the active H4K16ac histone mark [26] on the two hermaphrodites X chromosomes through the action of the dosage compensation complex (DCC) resulting in a global reduction in RNA Pol II recruitment [27]. On the other hand, in *D. melanogaster* (XX/XY system) the transcription of the single males’ X chromosome is doubled by an overall increase in the chromatin accessibility by the DCC [28, 29]. This complex is composed of five proteins: male-specific lethal 1, 2 and 3 (MSL1, MSL2 and MSL3), males absent on the first (MOF), maleless (MLE) and two lncRNAs (*roX1* and *roX2*) [29]. The DCC binds to the males X chromosome at several genomic locations where it modifies histones chemistry with the active histone mark H4K16ac [30, 31] that enhances the chromatin accessibility of the X in males compared to females, hence allowing an increased transcription of X-linked genes in males.

Aphids are major crop pests that can cause severe damages on a wide range of crops. Their success as pests is largely explained by their remarkable adaptive potential to their environment, and especially the phenotypic plasticity they display during their annual life cycle where they can switch from clonal to sexual reproduction [32, 33] in response to environmental cues. Viviparous parthenogenetic females reproduce clonally from spring to summer and, in response to photoperiod shortening at fall arrival, can generate sexual females and males that will mate and produce overwintering eggs [34–36]. The pea aphid *Acyrthosiphon pisum*—for which the genome is sequenced and partially assembled [37]—is a male heterogametic species with X0 males and XX females. A recent transcriptomic analysis of the different pea aphid sexual morphs by Jaquiéry et al. [34] revealed a general trend where X-linked genes are on average more transcribed in males than in females. Nevertheless, the global expression of X-linked and autosomal genes and consequently XX/AA, X/AA and XX/X ratios were not assessed at that time. Here, our study first aimed at addressing this point taking benefits from a much larger set of genes—being generated overtime [38]—assigned as X-linked or autosomal. We report that expression profiles of expressed X-linked and autosomal genes yield X/AA and XX/AA ratios close to 1, suggesting a chromosome-wide regulation of the expression of X-linked genes, and consequently a potential differential X chromosome chromatin accessibility between sexes. To test this hypothesis, we applied the FAIRE (Formaldehyde-Assisted Isolation of Regulatory Elements) methodology to extract and then sequence the open chromatin from whole male and parthenogenetic female (here after termed as “females”) individuals [39, 40]. This open chromatin mainly corresponds to nucleosome-depleted regions (NDR) likely to be more accessible to transcriptional (RNA polymerase II) or regulatory elements (mainly enhancers or insulators). Statistical analyses revealed that the transcription start sites (TSSs) from expressed genes were significantly more accessible on the X chromosome in males compared to females. More specifically, X-linked genes expressed at the same level in males and females and, thus likely to be compensated, have significantly higher chromatin accessibility in males than in females, notably in the TSS. These results suggest that a potential global regulation of chromatin accessibility might occur on the X chromosome of aphids to compensate for the gene dose in males.

## Results

### Expression of X-linked and autosomal genes in males and females

A new assignation of the genomic scaffolds of the *A. pisum* genome to autosomes and X chromosome has recently been performed [38]. We thus reanalyzed previously published *A. pisum* RNA-seq data from whole-individual males and parthenogenetic females (and to a lesser extent, sexual females, since FAIRE has been performed on asexual females and males; see the next result part concerning the FAIRE for a more in-depth explanation) [34] in regard to this new gene assignation to characterize the expression level of the 19,232 and 13,711 genes located on the X and autosomes, respectively. X/A expression ratios were calculated at different expression levels thresholds [15–17] (Table 1). When all genes are taken into account, XX/AA and X/AA ratios close to 0 are observed since over 80% of the X-linked genes are weakly or not expressed in both males and asexual females. When increasing the minimum expression levels thresholds, males X/AA ratio increases and approaches 1 when genes with a mean male and female RPKM (considered as mean RPKM hereafter) superior to 2 are considered. Above this expression level threshold, X-linked and autosomal genes are evenly expressed in males (Wilcoxon rank sum tests with *p* > 0.05). On the other hand, asexual females XX/AA ratios are in all cases lower than 1 (ranging from 0.45 to 0.69, *p* < 10^−16^ in all cases), suggesting that females autosomal genes are in average more expressed than X-linked genes.

**Table 1:** Ratio of expression between X chromosome(s) and autosomes for males and females using minimum RPKM threshold

Genes taken into account	Number of genes retained		Female		Male	
	X	A	XX/AA ratio	*p* value*	X/AA ratio	*p* value*
All genes	13,708	19,230	0.00	<2.20E−16	0.00	<2.20E−16
Genes with RPKM >1	1886	9087	0.45	<2.20E−16	0.83	1.16E−04
Genes with RPKM >2	1484	7871	0.58	<2.20E−16	0.94	0.372
Genes with RPKM >3	1260	7010	0.62	<2.20E−16	0.99	0.891
Genes with RPKM >4	1069	6275	0.69	<2.20E−16	0.91	0.119

All genes or different mean RPKM cutoffs have been considered to filter expressed genes

* *p* values were calculated using Wilcoxon rank sum tests to compare X-linked and autosomal genes expression for a given morph

**Fig. 2:**
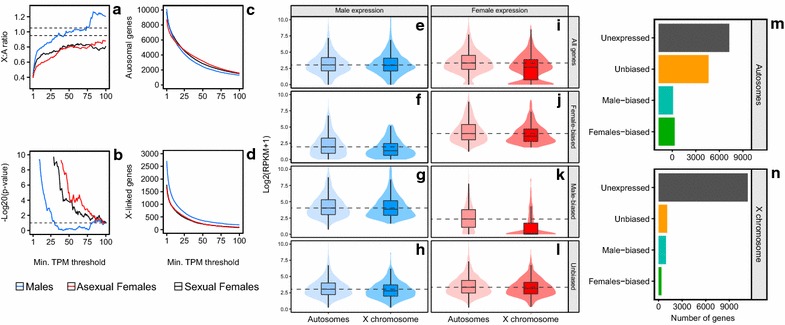
Expression data analysis to investigate dosage compensation in *Acyrthosiphon pisum*. **a**–**d** Comparison of the X-linked and autosomal genes transcription in males and both asexual and sexual females using increasing minimum TPM threshold to filter expressed genes. **a** The X/AA and XX/AA expression ratio of males (*blue*) and both asexual (*red*) and sexual (*black*) females, respectively. The *dashed lines* represent the dosage compensation criteria (X/A ratio between 0.95 and 1.05). **b** −Log20 (*p* value) of the Wilcoxon rank sum test comparing the expression of X-linked and autosomal genes. The *dashed line* represents *p* value = 0.05; all values below that line correspond to not significant Wilcoxon rank sum test. **c**, **d** Number of analyzed genes of A and B considering the expression filtration. Filters corresponding to dosage compensation for males are highlighted in *gray*, i.e., Wilcoxon rank sum test *p* value >0.05 and X/A ratio between 0.95 and 1.05. **e–l** Distribution of expression of autosomal and X-linked genes. Expression is represented for *all* (**e**, **i**), *female*-*biased* (**f**, **j**), *male*-*biased* (**g**, **k**) and *unbiased* (**h**, **l**) genes for males (*blue*) and females (*red*). These classes of expression have been determined using EdgeR (see “Methods”). The violin plots represent the gene density at each log2(RPKM + 1) level. *Dashed lines* correspond to the median of expression of autosomal gene in each category. **m**, **n** Number of genes belonging to each gene class on the X chromosome and on the autosomes

Similar results were obtained using an increasing minimum transcripts per million (TPM) filter separately in sexual females, asexual females and males, ranging from a minimum TPM of 1 to 100 (Fig. 2a–d). The X/AA ratio of males quickly goes up to 0.8, when very lowly expressed genes are filtered on both autosomes and the X chromosome (Fig. 2a). Dosage compensation for males, i.e., when their X/AA ratio is between 0.95 and 1.05 for Wilcoxon rank sum tests with *p* > 0.05), is attained with a minimum TPM threshold of 34–74 (overshadowed in gray, Fig. 2a, b). Both asexual and sexual females share a very similar expression pattern with a maximum XX/AA expression ratio of 0.8 (Fig. 2a) and with Wilcoxon rank sum tests that are always significant (*p* < 0.05), meaning that the distribution of expression of X-linked and autosomal genes is significantly different, despite the two X chromosomes of females (Fig. 2b).

To further investigate the uneven expression of X-linked and autosomal genes in the females, we classified genes (with mean RPKM >2 in males or asexual females) into four non-exclusive classes (*all*, *female*-*biased*, *male*-*biased and unbiased* genes) based on differential expression analysis (see “Methods,” Fig. 2) between males and asexual females (referred to as females hereafter), since FAIRE has been performed on these two sexes. When considering *all* genes (Fig. 2i), violin plots for females display a large group of lowly expressed genes on the X chromosome that pulls down the females’ median expression of X-linked genes. This lowly expressed gene group on the females X corresponds to *male*-*biased* genes (Fig. 2k), and when those are not taken into account, such as in the *unbiased* gene class (Fig. 2l), the XX/AA ratio is approaching 1 (ranging from 0.79 to 0.92 for genes with a mean RPKM >1–4). This suggests an almost balanced transcription in the females of the X-linked and autosomal *unbiased* genes, although the difference in expression between chromosomes is still significant (Wilcoxon rank sum test, *p* ranging from 7.10^−7^ to 0.03 for genes with a mean RPKM >1–4). Such group of lowly expressed genes is absent in males (Fig. 2e–h), thus leading to an equal expression of X-linked and autosomal genes for *all* (Fig. 2e) and *unbiased* (Fig. 2h) gene classes. Moreover, the number of each gene class on autosomes and on the X (Fig. 2m, n, respectively) reveals that *male*-*biased* genes are almost three times more represented than *female*-*biased* genes on the X while even numbers are observed on the autosomes for these two classes. This further explains the fact that the expression of the X chromosome in females is driven down compared to autosomes, and thus the 0.8 XX/AA ratio. When taking the results of both males and females into account, our analyses show that the XX/AA and X/AA ratios are approaching 1, especially for *unbiased* genes (Fig. 2h, l). Since males carry only one X per cell, and females two, these results suggest a global readjustment of X-linked genes transcription in males or females.

### Raw FAIRE-seq data analysis

To compare chromatin accessibility in males and females, we used the Formaldehyde-Assisted Isolation of Regulatory Elements (FAIRE) procedure to extract the DNA associated with NDR from whole individuals and for three pools of males and parthenogenetic females [39, 40], as well as a pool of control DNA for each sex. FAIRE experiments on sexual females were unsuccessful, which might be related to the important quantity of yolk contained in the eggs, interfering with the FAIRE procedure. We thus focused on asexual females and males for the FAIRE experiment. An average FAIRE ratio (measured as in 36) of 1.43% (±0.54) and 2.96% (±0.63) was obtained for males and asexual females, respectively (referred to as females hereafter). After sequencing the six FAIRE and the two Control DNA samples, FAIRE and Control Reads were mapped onto pea aphid genome and filtered to only conserve uniquely mapped reads.

The coverage of autosomes and X chromosomes was assessed in the control DNA libraries for both males and females (Additional file 1) yielding 14.3 million and 28.0 million 100-bp paired-end uniquely mapped reads for females and males, respectively. The female control library showed an equal coverage for autosomes and X chromosome which is expected since the female is diploid at both X chromosome and autosomes. In males—that are diploid for autosomes and haploid for the X chromosome—autosomes display an expected twofold higher coverage than the haploid X chromosome in males.

**Fig. 3:**
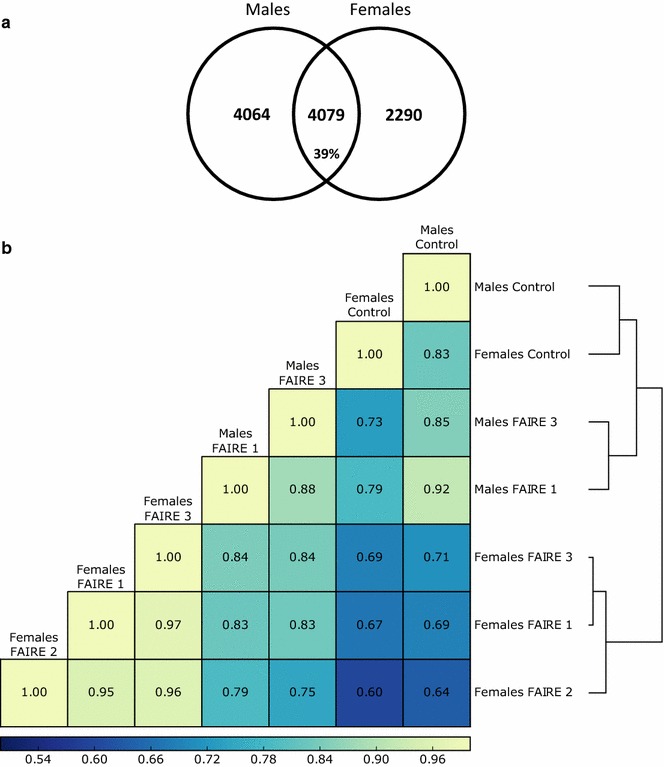
FAIRE peaks obtained after MACS2 and IDR analyses and clustering of FAIRE and Control libraries. **a** Number of FAIRE peaks in both males and females (total of 8143 and 6369, respectively). Their specificity between the two sexes is represented in the Venn diagram. **b** Clustering of the FAIRE and Control samples based on Pearson’s correlations. Correlation coefficients are shown and were calculated from the reads coverage of 10-bp bins along all open chromatin regions retained by MACS2 followed by IDR. The Males FAIRE 2 library is not included since it has been discarded by IDR because of its low reproducibility compared to Males FAIRE 1 and 3

The reproducible FAIRE biological replicates for males and females were pooled according to their reproducibility using the MACS2 peakcaller [41] followed by irreproducible discovery rate (IDR) [42, 43] peaks ranking analysis. For males, these algorithms resulted in the discrimination of one of the three male replicates as lowly correlated with the other two. After pooling the relevant FAIRE replicates, three female and two male libraries were then conserved which ended up with 20.5 million and 23.6 million 100-bp paired-end reads mapping to unique positions in the genome for female and male pools, respectively. MACS2 [41] and IDR [42] also allowed us to retain a set of reproducible FAIRE peaks across the biological replicates for each sex: 8143 FAIRE peaks for males and 6369 FAIRE peaks for females were then identified; 39% of these are overlapping between males and females and are thus non-sex-specific (Fig. 3a). In order to assess the level of correlation between the control and retained FAIRE replicates, we concatenated these peaks coordinates into a set of 10,433 FAIRE peaks using BEDTools [44] and performed a Pearson correlation and a hierarchical clustering using deepTools2 [45] (Fig. 3b). Control libraries are alike (Pearson’s *R* ^2^ of 0.83) and segregate together. Female and male FAIRE libraries are separated in the hierarchical clustering, and Pearson’s *R* ^2^ values within each sex are high: 0.88 for males and more than 0.95 for female libraries comparisons.

**Fig. 4:**
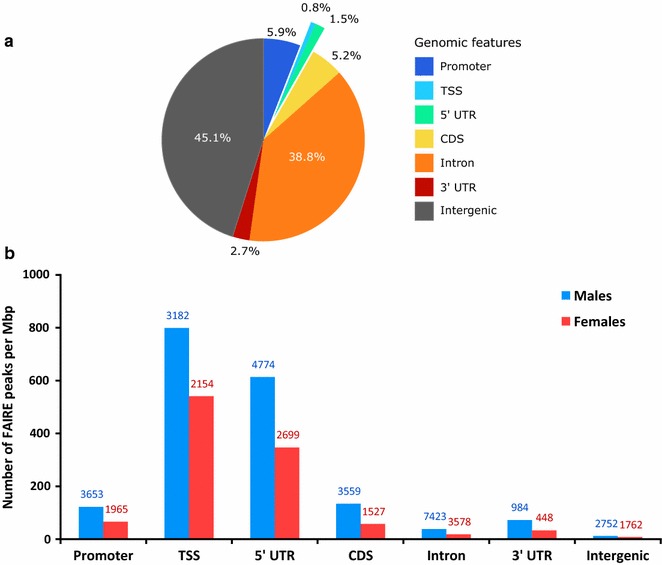
FAIRE peaks density for the different genomic features of *A. pisum*. **a** Overlapping of the identified FAIRE peaks using MACS2 and IDR with the various genomic features of *A. pisum* genome annotation v2.1 expressed as the number of peaks per million of base pairs (peaks per Mb) for male (*blue*) and female (*red*) individuals. The raw number of peaks is *indicated above each bar*. The sum of the raw FAIRE peaks is not equal to the total peaks presented in 2. A since one peak can overlap multiple genomic features. **b** Percentage of the different genomic features in the pea aphid genome annotation (aphidbase v2.1)

To describe the overall distribution of the identified FAIRE peaks across the genome, we calculated the abundance of FAIRE peaks overlapping with different genomic features (promoters, TSSs, UTRs, exons, introns and intergenic regions) (Fig. 4). 5′UTRs and TSSs are characterized by high FAIRE peaks densities (Fig. 4a): Although these genomic features represent, respectively, only 1.5 and 0.8% of the genome (Fig. 4b), they contain a large proportion of FAIRE peaks compared with the other genomic features that represent larger parts of the genome. The FAIRE peaks density is thus much lower in all other genomics features (Fig. 4a). Examples of genomic regions displaying some of these open chromatin peaks specific of each sex or in common between sexes near sex-specific or housekeeping genes are observable in Additional file 2.

### Open chromatin signal in males and females

**Fig. 5:**
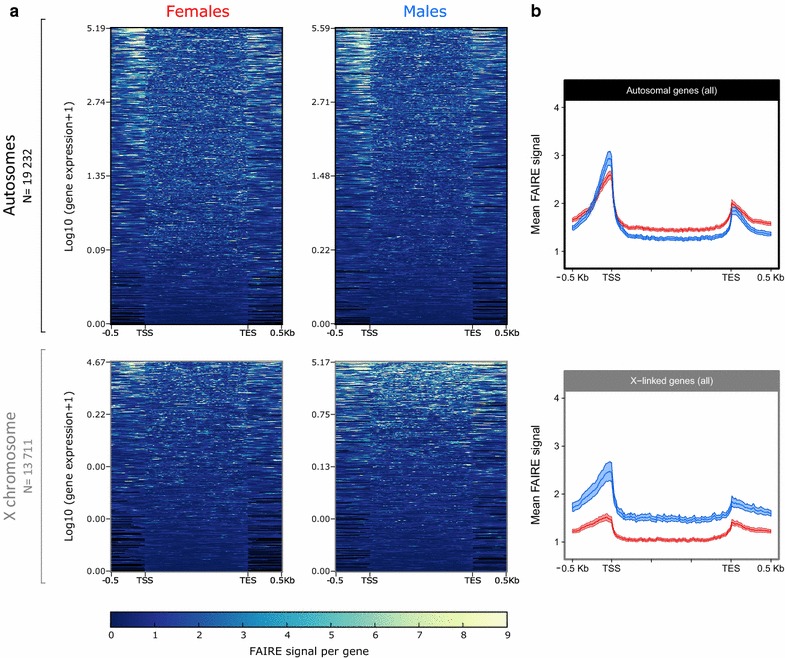
FAIRE signal profiling of *A. pisum* females and males for all autosomal and X-linked genes. The input normalized FAIRE signal has been calculated by dividing FAIRE reads by Control reads in bins of 10 bp around (500 pb upstream the TSS and 500 bp downstream the TES) and in the genes body that all have been equalized to 1500 bp. **a** FAIRE signal is shown for every gene carried by the autosomes (*top*, *black*) or by the X chromosome (*bottom*, *gray*). Genes are ranked from the most expressed to the least expressed depending on the sex and on the chromosome type. **b** Mean FAIRE signal calculated for all autosomal genes (*top*, *black*) and X-linked genes (*bottom*, *gray*) for males (*blue*) and females (*red*). 99% CI based on 1000 bootstrap is shown

To assess the pea aphid’s chromatin opening profile, we calculated the FAIRE signal at the genome scale by dividing the normalized FAIRE coverage by the normalized Control coverage on 10-bp windows (hereafter named bins, as described by deepTools [45]). In Fig. 5a, the FAIRE signal is represented for each gene of the pea aphid genome and RNA-seq data have been used to rank genes according to their level of expression in males or in females. The strongest FAIRE signals are observed in regions upstream the TSS. The more a gene is expressed, the more its FAIRE signal upstream the TSS is high, indicating a positive correlation between gene expression and chromatin accessibility. The heatmaps representing the FAIRE signal along X-linked genes suggest a stronger FAIRE enrichment in males than in females, in comparison with the autosomes that share similar profiles between sexes (Fig. 5a).

We then calculated the mean genic FAIRE signal and 99% confidence interval (CI) for both males and females on autosomal and X-linked genes (Fig. 5b). According to the previous observation, autosomal genes share similar accessibility levels in males and females, with a slightly higher accessibility near the TSS in males than in females based on 99% CI (2.94 ± 0.13 in males and 2.59 ± 0.09 in females on the most accessible bin). Contrastingly, the mean FAIRE signal along and around X-linked genes is almost two times higher in males than in females, especially near the TSS (2.40 ± 0.18 for males and 1.45 ± 0.07 for females on the most accessible bin). In order to verify whether the FAIRE signal normalization on Control DNA could introduce a bias for the X chromosome, we calculated the mean FAIRE coverage normalized by sequencing depth around all autosomal and X-linked genes in males and females (Additional file 3). It appears that genes on the autosomes and on the X chromosome are equally accessible between males and females, despite the single X in males compared to the two X chromosomes of females. This profile does not invalidate the results observed using input normalized FAIRE signal, since the single X chromosome of males is still more accessible than each female X chromosome, and is thus differentially accessible.

**Fig. 6:**
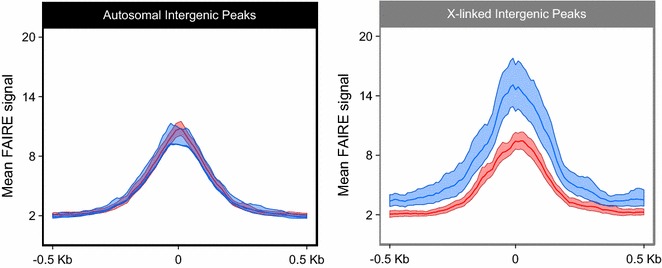
Mean FAIRE signal of X-linked and autosomal intergenic peaks. The input normalized FAIRE signal has been calculated around intergenic FAIRE-enriched peaks identified using MACS2 and IDR for males (*blue*) and females (*red*) on the autosomes (*black*) and X chromosome (*gray*). Overlapping peaks are enriched FAIRE regions in common between males and females, while sex-specific FAIRE peaks do not show any overlap between the two morphs

We then investigated the FAIRE signal within intergenic regions in males and females. The assessment of the chromatin accessibility in these intergenic regions was performed around the summit of the intergenic FAIRE peaks retained by IDR. FAIRE peaks that do not overlap any annotation feature were thus extracted and defined as intergenic FAIRE peaks. The mean FAIRE signal and 99% CI were calculated 1000 bp around the summits of those intergenic FAIRE peaks for males and females (Fig. 6). On autosomal intergenic FAIRE peaks, no significant differences of chromatin accessibility were identified between males and females. Contrastingly, for the X chromosome, intergenic FAIRE peaks in males are significantly more accessible than in females, especially near the peaks summits (mean FAIRE signal of 14.90 ± 2.46 for males and 9.35 ± 0.82 for females on the most accessible bin). The results for intergenic regions are thus similar to those observed for genic regions and demonstrate a comparable accessibility of the autosomes in the two sexes and an increased accessibility of the single X in males compared to the two X chromosomes in females.

### Link between genes expression and chromatin accessibility

**Fig. 7:**
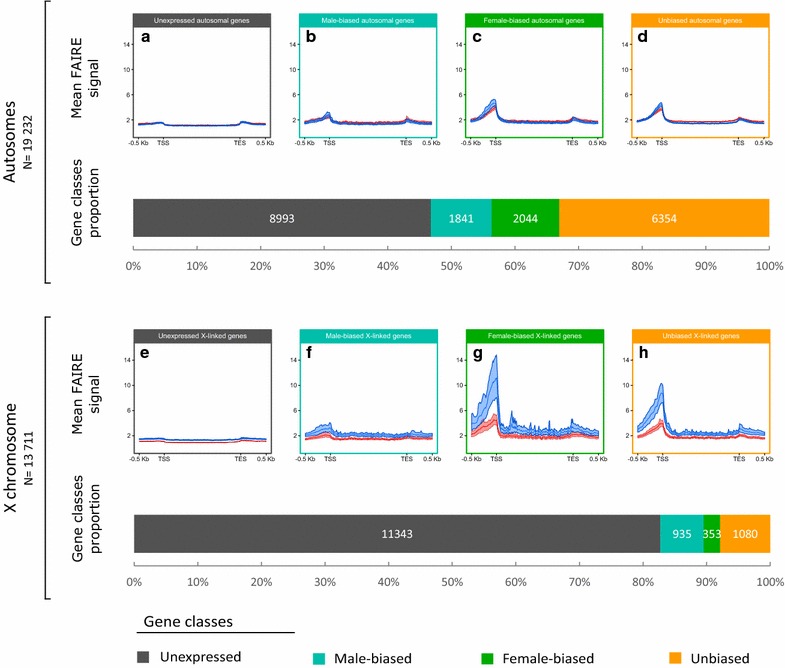
Mean FAIRE signal and proportion of X-linked and autosomal genes depending on their expression profile, grouped by chromosome type. Genes have been categorized into four different classes: *unexpressed* (**a**, **e**), *male*-*biased* (**b**, **f**), *female*-*biased* (**c**, **g**) and *unbiased* (**d**, **h**) genes between the two sexes based on gene expression data. The mean input normalized FAIRE signal has been calculated around (500 bp) each gene class and in the genes body (scaled) depending on the sex (males in *blue* and females in *red*) and on the chromosome type (autosomes, *top* and X chromosome, *bottom*). 99% CI based on bootstraps is also shown around the mean. The proportion and the number of the four gene classes are represented for autosomes and for the X chromosome. The same results grouped by sex instead of chromosome type are available as Additional file 4

To explore the link between gene expression and chromatin accessibility, and especially around the TSSs, we grouped genes into four classes depending on their expression patterns in males and females and studied their FAIRE signal profile considering both sexes and chromosome type (Fig. 7): *unexpressed* (Fig. 7a, e) (46.8% on the autosomes, 82.7% on the X), *male*-*biased* (Fig. 7b, f) (9.6% on the autosomes, 6.8% on the X), *female*-*biased* (Fig. 7c, g) (10.6% on the autosomes, 2.6% on the X) and *unbiased* genes (Fig. 7d, h) (33.0% on the autosomes, 7.9% on the X). These classes correspond to the ones defined during the RNA-seq experiment (Fig. 2). On the autosomes, *unexpressed*, *male*-*biased* and *female*-*biased* gene classes share a similar chromatin accessibility profile in males and females (Fig. 7a–c), and only *unbiased genes* (Fig. 7d) are slightly more accessible in the males TSS compared to females (4.41 ± 0.32 in males and 3.68 ± 0.22 in females on the most accessible bin). Contrastingly, on the X chromosome (Fig. 7e–h), two main profiles can be distinguished. The first group is comprised of *unexpressed* and *male*-*biased* genes (Fig. 7e, f) that display comparable—yet significantly different—FAIRE signal in males and in females: 1.56 ± 0.09 for males and 1.17 ± 0.05 for females regarding *unexpressed* genes and 3.27 ± 0.72 for males and 2.05 ± 0.33 for females concerning the *male*-*biased* genes. The second group is composed of *female*-*biased* and *unbiased* genes (Fig. 7g, h) that display a stronger chromatin accessibility difference between sexes. Mean FAIRE signals of 10.72 ± 3.06 in males and 4.41 ± 0.84 in females are observed for *female*-*biased genes* and those of 8.80 ± 1.55 in males and 3.95 ± 0.52 in females are observed for *unbiased genes*. These results suggest that *unbiased* and *female*-*biased* genes participate in majority to the observed global enhanced accessibility of the X chromosome in males compared to females (Fig. 5), even if they represent only 10% of X-linked genes. Interestingly, we can also observe that in females for *unbiased* (Additional file 4H) and *female*-*biased* (Additional file 4G) gene classes, chromatin accessibility is similar between the X chromosome and autosomes. On the contrary in males and for the same gene classes, chromatin accessibility is higher on the X chromosome than on autosomes (Additional file 4C, D). This suggests that autosomal genes in males and females and X chromosome in females share similar chromatin patterns and that the differential X chromosome chromatin pattern between sexes is explained by an enhancement of chromatin accessibility in males rather than a reduced accessibility in females.

## Discussion

In this study, we first reanalyzed whole-body RNA-seq data of pea aphid males and females and evidenced a potential dosage compensation mechanism. We then generated an overview of the open chromatin structure (nucleosome-depleted regions) of whole-body pea aphid males and females using the Formaldehyde-Assisted Isolation of Regulatory Elements. This first genome-wide epigenetic study in aphids demonstrated an enhanced chromatin accessibility of the males’ single X chromosome compared to the two X of the females.

### Global transcriptomic profiles suggest potential dosage compensation in pea aphid males

Using a total of 3712 genes assigned to autosomes or the X chromosome, Jaquiéry et al. [34] outlined that in the pea aphid, autosomal genes display similar transcription levels between males and females while X-linked genes are slightly more expressed in males than in females. Based on the same data, Pal and Vicoso [46] outlined different results as they found that males and females share similar expression levels on both X and autosomes. These contradictory results can, however, be explained by a misassignment of genes to chromosomes due to a widespread scaffold misassembly in the pea aphid genome [38]. Here, we used a new assignation of scaffolds to chromosomes [38] to analyze X-linked and autosomal gene expression patterns in females and males.

In males, we showed X/AA expression ratios almost equal to 1 when taking into account consistently expressed genes (mean RPKM >2). Similar expression patterns in organisms showing complete dosage compensation have been demonstrated such as in *Manduca sexta* heads [17] where the Z/AA ratio is approaching 1 when lowly expressed genes are not considered. These results suggest a potential dosage compensation mechanism in the male pea aphids.

Contrastingly, the females XX/AA expression ratios are inferior to 1 which is not a common feature within male heterogametic species with complete dosage compensation. Nevertheless, such an unusual ratio can also be observed in the flour beetle (*Tribolium castaneum*). In this species, females display *a* XX/AA ratio superior to 1 which is explained by the fact that females show a global X-linked genes overexpression (supported by an enrichment of female-biased genes on the X), while males show a X/AA ratio equal to 1, ending up with a XX/X ratio different from 1 [47]. In the pea aphid, the X chromosome is predicted to be masculinized [34], and we indeed observed that X-linked *male*-*biased* genes are almost three times more represented than X-linked *female*-*biased* genes and that most of the X-linked *male*-*biased* genes are male specific (i.e., expressed in males but not at all in females, as shown in Fig. 2k). As a result, XX/AA female ratio and female-to-male XX/X ratio are inferior to 1. It has to be noted that mRNA extractions were performed on whole individuals and thus contain mRNAs from both somatic and germ cells. The fact that most X-linked *male*-*biased* genes are almost male specific suggests that these genes could be testis specific or more generally involved in male phenotypic traits. GO terms enrichment analyses on *female*-*biased*, *male*-*biased* and *unbiased* gene classes on the X and autosomes revealed that only *male*-*biased* and *female*-*biased* genes on the X display reproductive-related functions. *Male*-*biased* X-linked genes display an enrichment of biological processes related to male-specific reproduction such as cilium movement, microtubule-based movement, sperm competition and multi-organism reproductive process (Additional file 5). Because the pea aphid’s spermatozoids are flagellated [48], such functions are likely to correspond to testis-specific genes, thus reinforcing the hypothesis of their absence of expression in females because of their male germ cells specificity. The unusual females XX/AA ratio (<1) for a male heterogametic species can thus be explained and is compatible with complete dosage compensation in the male pea aphids.

In mammals and in Drosophila [20, 49], dosage compensation mechanism activity on the X chromosome can be different between germ cells and somatic cells. Mammals do not show any dosage compensation in the germ cells where the X chromosome inactivation in females is withdrawn in developing primordial germ cells, allowing X-linked genes expression in female gametes [20]. Contrastingly, *D. melanogaster* germ cells compensate for gene dose in germlines with, however, distinct mechanisms than the well-known somatic cells dosage compensation [49]. These studies suggest that dosage compensation does not always occur in germ cells. Since the RNA-seq data analyzed in our study comprise mRNAs from germ cells and somatic cells, we cannot conclude whether the potential dosage compensation in the male pea aphids occurs only in somatic cells or in both somatic and germ cells.

### Dosage compensation in the pea aphid might be achieved by an enhanced chromatin accessibility of the X chromosome in males

Dosage compensation is often controlled by epigenetic mechanisms mediating the X or Z chromosome chromatin accessibility. Since our RNA-seq analyses suggest potential complete dosage compensation in the male pea aphids, we aimed at assessing the global chromatin accessibility of the autosomes and the X chromosome(s) in both males and females using FAIRE-seq. The FAIRE methodology allows the identification of open chromatin (nucleosome-depleted) regions. It has been performed for the first time by Giresi et al. [39] and since then has been used in various organisms and tissues [39, 40, 50]. Methods such as assay for transposase-accessible chromatin (ATAC) [51–53] are becoming alternatives to the FAIRE overtime; however, the latter remains a reliable way to assess open chromatin regions at the genome scale when coupled to high-throughput sequencing.

To validate the FAIRE-seq approach used here, we checked the presence of a high FAIRE peaks frequency in TSS and 5′UTR regions, as expected since TSSs are known as the most accessible regions in the genome in order to allow the binding of the RNA polymerase II and thus the transcription of the gene [40, 54–56]. We also observed a positive correlation between genes expression and chromatin opening upstream their TSS, which corresponds to a typical profile of a successful FAIRE experiment [40].

With our FAIRE-seq approach, we then showed a similar mean chromatin accessibility of autosomes in males and females over all genes (Fig. 5b; Additional file 3) and also when genes are classified according to their expression patterns in males and females (Fig. 7; Additional file 4). Similar chromatin accessibility in males and females is also observed within open intergenic regions. These results suggest that the autosomes of the pea aphid are equally accessible in males and females.

Contrastingly, the FAIRE signal analysis over *all* genes (Fig. 5b) revealed that X-linked genes are more accessible in males than in females, or evenly accessible despite the single X of males using sequencing depth normalization alone (Additional file 3). *Female*-*biased* and *unbiased* X-linked genes (Fig. 7g, h) display the most important difference of chromatin accessibility between males and females and thus contribute at most to the global male-enhanced chromatin accessibility of the X chromosome, suggesting that the dosage compensation in males might be partial. Since *unbiased* genes have (by construction) similar expression pattern between males and females, X-linked *unbiased* genes correspond to potentially compensated genes. A higher chromatin accessibility of these genes in males than in females thus supports the hypothesis of potential epigenetic mechanism underlying dosage compensation in males. Surprisingly, X-linked *female*-*biased* genes chromatin accessibility is comparatively higher in males than in females (Fig. 7g). This cannot be used to reject this hypothesis since in *D. melanogaster* males, some female-biased genes also show an enrichment of the active histone mark H4K16ac mediated by the DCC, thus resulting in an enhanced chromatin accessibility for these genes [57]. Additionally, X-linked intergenic FAIRE peaks, which represent only a very small proportion of intergenic regions, are significantly more accessible in males than in females. Altogether, these data suggest a global regulation of the X chromosome chromatin accessibility in order to compensate for gene dose in males.

Interestingly, the chromatin accessibility of X-linked *male*-*biased* genes is only slightly enhanced in males, despite their high level of expression in that morph. As suggested by our GO analyses, these genes could correspond in majority to testis-specific genes (hence including germ cells-specific genes). The relatively low chromatin accessibility of these genes could be explained on the one hand by the fact that FAIRE is a population assay and that male-specific cells make up only a small percentage of the cell population in the analyzed whole-body male individuals. The FAIRE signal could thus be low for the particular class of *male*-*biased* genes, hence resulting in a low difference in FAIRE signal between males and females for that gene class. On the other hand, these results could suggest that the epigenetic mechanism underlying the male-enhanced X chromosome chromatin accessibility does not take place in male germ cells, like in the female mammals for example [20]. *Female*-*biased* and *unbiased* genes might then be part of a given X chromosome territory compensated for the lack of a second X chromosome in the males’ genome, while the *male*-*biased* and *unexpressed* genes might be part of another X chromosome territory where the chromatin accessibility is not submitted to a given mechanism underlying dosage compensation. Such hypothesis would resemble what has been identified in the Drosophila where only 75% of the X chromosome territory is regulated by the DCC [29].

The high chromatin accessibility of the male single X chromosome might be the consequence of an underlying epigenetic mechanism taking place either in male (enhanced chromatin accessibility) or in female individuals (reduced chromatin accessibility). When taking *unbiased* and *female*-*biased* gene classes into account (Fig. 7c, d, g, h), we can observe that autosomal genes in males and females as well as X-linked genes in females are equally accessible, whereas X-linked genes are more accessible only in males. The differential X chromosome chromatin accessibility observed between sexes can thus be explained by an augmentation of such accessibility in males rather than a reduction in females. This suggests the existence in pea aphids males of an epigenetic mechanism promoting the enhancement of the X chromosome chromatin accessibility associated with dosage compensation. These epigenetic patterns resemble the Drosophila model where an overall increase in chromatin accessibility is observed on the males X chromosome through a male-specific H4K16ac histone posttranslational modification enrichment mediated by the DCC in the gene bodies (H4K16ac being enriched in the genes’ TSS and promoter by the NSL complex) [28, 57]. Interestingly, the pea aphid genome contains protein coding for genes homologous to the five proteins composing the *D. melanogaster* DCC (Additional files 6, 7). The DCC is also constituted by two additional *roX* lncRNAs that were not found in the pea aphid genome, but it is noteworthy that lncRNA sequences are rarely conserved between organisms [58]. Interestingly, homologs identities and similarities between *D. melanogaster* and *A. pisum* DCC proteins are found particularly in functional domains. The most conserved proteic domains between the Drosophila and the pea aphid are MOF’s MOZ-SAS domain and all MLE’s proteic domains, namely dsrm, DEAD, helicase C, HA2 and OB NTP BIND (Additional file 7). MOZ-SAS domain from Drosophila’s MOF is involved in the acetylation of lysine 16 of histone 4 (H4K16ac) and is thus responsible for the enhanced chromatin accessibility along the Drosophila males X chromosome. The high proteic domain conservation observed between the pea aphid and the Drosophila could lead to conserved functions of the MOF and MLE proteins in these two organisms. However, functional analyses are required to validate this hypothesis. Moreover, some of the proteins composing the Drosophila’s DCC, including MOF and MLE, has been identified as conserved in mammals without, however, playing a role in dosage compensation [29, 59–63]. The pea aphid could then, independently of a DCC complex, display a different epigenetic mechanism supporting dosage compensation by globally enhancing chromatin accessibility of the single X chromosome in males, an alternative that cannot be ruled out taking into account the rather weak conservation of MSL2 protein sequence between Drosophila and the pea aphid (Additional file 7), particularly in the RING domain which plays a role in the DCC assembly [64].

## Conclusions

This study gives a first insight into *A. pisum* chromatin accessibility patterns in relation to a possible dosage compensation mechanism. The males single X chromosome is globally more accessible than the two female X chromosomes. More importantly, X-linked genes showing similar expression levels between females and males and that could potentially be compensated in the latter are more accessible in males. Further experiments—especially chromatin immunoprecipitation followed by high-throughput sequencing targeting specific histone marks—must be conducted in order to characterize the underlying epigenetic mechanism involved in this overall enhanced chromatin accessibility of the males single X chromosome.

## Methods

### Aphids rearing

*Acyrthosiphon pisum* individuals from the clone LSR1 (the reference clone that was used for genome sequencing [37]) were reared on broad bean *Vicia faba* at low density (less than five individuals per plant) to prevent the production of winged morphs for at least two generations. Parthenogenesis was maintained under a 16-h photoperiod and a temperature of 18 °C. At the third generation, 20 asexual females were then directly frozen into liquid nitrogen for FAIRE extraction. The production of male individuals was initiated by transferring larvae from a 16-h to a 12-h photoperiod at the same temperature of 18 °C [34]. Two generations later, males were produced. A total of 100 adult males were then directly frozen into liquid nitrogen for FAIRE extraction. No RNA extraction was performed since already published RNA-seq data were used [34].

### RNA high-throughput sequencing

We reanalyzed six RNA-seq libraries used by Jaquiéry et al. [34]. Briefly, these six libraries correspond to three male libraries and three parthenogenetic female libraries of clone LSR1. Details regarding aphid rearing, RNA extraction, libraries preparation and sequencing are provided in Jaquiéry et al. [34]. Libraries were mapped on the version 2 of the pea aphid genome assembly using TopHat2 (RRID:SCR_013035) default parameters [65]. The number of reads covering each CDS of the gene prediction v2.1 was then counted using HTSeq-count (RRID:SCR_011867) [66] with the following parameters: –m intersection-strict –s no –t exon. The numbers of mapped reads per library ranged from 7.3 to 20 million, with an average over libraries of 14.3 million reads. Raw read counts were normalized in R (RRID:SCR_001905) [67] with edgeR (RRID:SCR_012802) [68] package by sequencing depth using the TMM method and by genes length (RPKM and TPM calculation). Genes were filtered using increasing minimum TPM threshold ranging from 1 to 100 using a step of 1 in order to remove the less expressed genes. Within each TPM filtration step, X/A ratio in males and females, Wilcoxon rank sum test *p* value and the number of retained X-linked and autosomal genes have been calculated. Differential expression between males and parthenogenetic females for each gene was tested with edgeR, considering the different libraries for each morph as replicates using genewise exact tests for differences in the means between two groups of negative-binomially distributed counts, based on normalized read counts. Genes have then been grouped into four classes: *unexpressed*, *male*-*biased*, *female*-*biased* and *unbiased* genes. *Unexpressed* genes have been determined as such if they displayed less than 1 count per million in at least three libraries as in [69]. *Male*-*biased* and *female*-*biased* genes have been determined as such using a FDR <0.05. *Unbiased* genes comprise differentially expressed genes with a FDR >0.05 and genes non-differentially expressed.

### Formaldehyde-Assisted Isolation of Regulation Elements, sequencing and bioinformatics analyses

The FAIRE extraction of three frozen pools of male and parthenogenetic female individuals was performed following the protocol of frozen tissues proposed by Simon et al. [40] with the following parameters. Tissues were first ground using a Biospec Bio-pulverizer and then fixed by the addition of 3% of Thermo Scientific Pierce formaldehyde during 8 min. The fixation was then stopped by the addition of glycine at 125 mM. Subsequently to the pellet rinsing and resuspension, the tissues were ground with a Tissue Lyser, using Qiagen metallic beads, during five cycles of 5 min, each being interrupted during 2 min. The sonication steps were performed using a Bioruptor Plus during 12 cycles of 30 s, each being interrupted during 30 s. Phenol chloroform extraction steps were performed using Sigma phenol chloroform. Subsequently to its extraction, the FAIRE DNA was purified the using ZYMO ChIP DNA Clean & Concentrator. Finally, FAIRE and Control DNA were quantified using a Quantus with the Quantifluor dsDNA kit. The reproducibility of the replicates was assessed before sequencing by calculating the FAIRE/Control ratio described in Simon et al. [40].

FAIRE and Control DNA were sequenced using the Illumina Hiseq 2000 instrument and ChIP TrueSeq kit. Three FAIRE samples for each morph were sequenced, while only a pool of the three samples from the same morph individuals was sequenced for control DNA. The eight different samples (six FAIRE and two Control samples) were 100-pb paired-end sequenced on a single lane. The raw sequenced data for these eight samples are available online at the NCBI under the BioProject accession number PRJNA348188. The reads were mapped using bowtie2 with default parameters [70, 71]. Only uniquely mapped reads with a mapping quality over or equal to 30 were kept using SAMtools (RRID:SCR_002105) [72], following the IDR recommendations [42, 43]. For peak calling, we analyzed separately the reads mapping on the X and on autosomes to avoid bias related to the difference of coverage of the X chromosome (haploid in males) and autosomes (diploid in males and females). In addition, Jaquiéry et al. [38] showed that over 50% of scaffolds greater than 150 kb are chimeras of X and autosomes, which could induce artificial peaks at breakpoints. Following IDR recommendations, MACS2 (RRID:SCR_013291) [41] was used to perform the peak calling with the following parameters: –f BAM –nomodel –extsize 200 –slocal 1000 –llocal 10,000 –*p* 0.05. The –gsize parameter (which corresponds to the total size of the genome analyzed) has been adapted depending on the size of chromosome types (166,018,072 bp for the X and 340,086,956 bp for autosomes). IDR analyses were then performed as suggested in [43] using a threshold of 0.04 for original replicates, of 0.04 for self-consistency replicates and of 0.01 for pooled pseudoreplicates. Once the final peak set for males and females was generated, HOMER suite (RRID:SCR_010881) [73] was used to calculate Venn Diagrams of males and females FAIRE peaks specificity. Segtools (RRID:SCR_004394) [74] with the aphidbase (RRID:SCR_001765) version 2.1 annotation of the pea aphid genome were used to assign the FAIRE peaks to various genomic features such as promoters (1300 bp upstream TSS regions), TSS (defined as 200 bp upstream the first base of 5′UTRs), 5′UTR, coding regions, introns, 3′UTR and intergenic regions. DeepTools2 [45] was used to calculate and represent the FAIRE signal as the ratio between pooled FAIRE reads over Control reads on bins of 10 pb on whole genome. FAIRE signal data were first retrieved along every gene and represented in heatmaps with deepTools2 [45]. The mean FAIRE signal was calculated using R and represented using the ggplot2 package [67, 75]. In order to estimate a confidence interval for each mean FAIRE signal calculated, bootstrap was done using a custom R script that performed 1000 random resampling of the genes by taking into account the number of genes in each resampled set. 99% confidence interval, which depends on gene number and FAIRE signal values, was then calculated and represented using the ggplot2 [75] package in the R software [67].

### Drosophila BLAST and GO terms enrichment of molecular processes in the pea aphid

Since *A. pisum* gene functions are poorly characterized, the most efficient way to perform GO terms enrichment in this organism was to compare unique *D. melanogaster* homologs of a given list of pea aphid genes of interest against all the unique *D. melanogaster* homologs found in the pea aphid annotation (aphidbase v2.1). In order to find *D. melanogaster* homologs in the pea aphid, BLASTp (RRID:SCR_001010) [76] analyses were performed using BLAST+ [77] with default parameters. GO terms enrichment of biological processes has then been done using GOrilla (RRID:SCR_006848) with default parameters [78, 79] using unique *D. melanogaster*’s homologs coming from a gene list of interest as “target set” and all the unique *D. melanogaster* homologs found in the pea aphid annotation v2.1 as “background set”.

## Additional files

**Additional file 1.** FAIRE and Control libraries coverages. Number of reads and coverage of X chromosome (X) and autosomes (A) for male and female control libraries.

**Additional file 2.** Genome browser view of remarkable autosomal and X-linked regions displaying sex-specific and non-specific FAIRE-seq and RNA-seq signal. **A**, **D**: female-specific regions around the genes ACYPI003071 (uncharacterized protein) and ACYPI001644 (cuticular protein 44). **B**, **E**: male-specific regions around the genes ACYPI080359 (uncharacterized protein) and ACYPI081672 (uncharacterized protein). **C**, **F**: regions in common between males and females for the genes ACYPI000061 (ATP synthase subunit beta) and ACYPI006656 (molybdate-anion transporter). The RNA-seq and FAIRE-seq signals have been made equal between males and females for each region.

**Additional file 3.** Mean FAIRE coverage calculated for all autosomal genes (left, black) and X-linked genes (right, gray) for males (blue) and females (red). 99% CI based on 1000 bootstrap is shown around the mean. The FAIRE coverage has been normalized by read-depth in order to allow the comparison between males and females data.

**Additional file 4.** Mean FAIRE signal and proportion of X-linked and autosomal genes depending on their expression profile, grouped by sex. Genes have been categorized in four different classes: *unexpressed* (**A**, **E**), *male*-*biased* (**B**, **F**), *female*-*biased* (**C**, **G**) and *unbiased* (**D**, **H**) genes between the two sexes based on gene expression data. The mean FAIRE signal has been calculated around each gene class (500 bp) and in their gene body (scaled), depending on the chromosome type (autosomes in dark color, X chromosome in light color) and depending on the sex (males, top in blue and females, bottom in red. 99% CI based on bootstraps is also shown around the mean.

**Additional file 5.** GO enrichment of biological processes for autosomal and X-linked, male- and female-biased genes.

**Additional file 6.** Protein sequence comparison of the Drosophila’s DCC in the pea aphid. Clustal multiple sequence alignment by MUSCLE (3.8) of *A. pisum*’s homologs of *D. melanogaster* DCC. Proteic domains of the Drosophila are represented in full color boxes above the alignments. *A. pisum* gene names found by BLASTp are indicated in parenthesis.

**Additional file 7.** Protein conservation of the Drosophila’s DCC in the pea aphid. Proteic domains percentage of identity between *A. pisum* homologs of the five proteins composing the DCC of *D. melanogaster.*

## Abbreviations

H3K4me1: monomethylated lysine 4 of histone 3
H3K9ac: acetylated lysine 9 of histone 3
macroH2A1: histone variant macroH2A1
H3K27me3: trimethylated lysine 27 of histone 3
lncRNA: long noncoding RNA
H4K20me1: monomethylated lysine 20 of histone 4
H4K16ac: acetylated lysine 16 of histone 4
DCC: dosage compensation complex
RNA Pol II: RNA polymerase II
MSL1, MSL2 and MSL3: male-specific lethal 1, 2 and 3
MOF: males absent on the first
MLE: maleless
*roX1*, *roX2*: RNA on X chromosome 1 or 2
FAIRE: Formaldehyde-Assisted Isolation of Regulatory Elements
NDR: nucleosome-depleted regions
TSS: transcription start site
RPKM: reads per kilobase of transcript per million mapped reads
TPM: transcripts per million
IDR: irreproducible discovery rate
MACS2: model-based analysis for ChIP-seq
5′/3′UTR: five or three prime untranslated region
CI: confidence interval
GO: gene ontology
ATAC: assay for transposase-accessible chromatin
MOZ-SAS: MOZ is a monocytic leukemia Zn_finger protein and the SAS protein from *Saccharomyces cerevisiae*
FDR: false discovery rate
ChIP: chromatin immunoprecipitation
HOMER: Hypergeometric Optimization of Motif EnRichment
BLAST: basic local alignment search tool
NCBI: National Center for Biotechnology Information
MUSCLE: MUltiple Sequence Comparison by Log-Expectation
