# Supplementary material for: Dosage compensation and sex-specific epigenetic landscape of the X chromosome in the pea aphid
Source: Epigenetics Chromatin. 2017 Jun 15;10:30. doi: 10.1186/s13072-017-0137-1 (PMC5471693; doi:10.1186/s13072-017-0137-1)
Supplement: Supplementary file 1 — Additional file 1. FAIRE and Control libraries coverages. Number of reads and coverage of X chromosome (X) and autosomes (A) for male and female control libraries. [file 13072_2017_137_MOESM1_ESM.pdf]

Additional file 1: FAIRE and Control libraries coverages. Number of reads and coverage of X chromosome (X) and autosomes (A) for male and female control libraries.

	Female Control (AA XX)		Male Control (AA X0)	
	X	A	X	A
Number of reads	4 906 829	9 454 084	5 304 035	22 708 453
Number of base covered	490 682 900	945 408 400	530 403 500	2 270 845 300
Size	166 018 072	340 086 956	166 018 072	340 086 956
**Coverage (expressed in x)**	**3.0**	**2.8**	**3.2**	**6.0**
