# Supplementary material for: Dosage compensation and sex-specific epigenetic landscape of the X chromosome in the pea aphid
Source: Epigenetics Chromatin. 2017 Jun 15;10:30. doi: 10.1186/s13072-017-0137-1 (PMC5471693; doi:10.1186/s13072-017-0137-1)

Additional file 2: Genome browser view of remarkable autosomal and X-linked regions displaying sex-specific and non-specific FAIRE-seq and RNA-seq signal. A, D: female specific regions around the genes ACYPI003071 (uncharacterized protein) and ACYPI001644 (cuticular protein 44). **B, E**: male specific regions around the genes ACYPI080359 (uncharacterized protein) and ACYPI081672 (uncharacterized protein). **C, F**: regions in common between males and females for the genes ACYPI000061 (ATP synthase subunit beta) and ACYPI006656 (molybdate-anion transporter). The RNA-seq and FAIRE-seq signals have been made equal between males and females for each region.

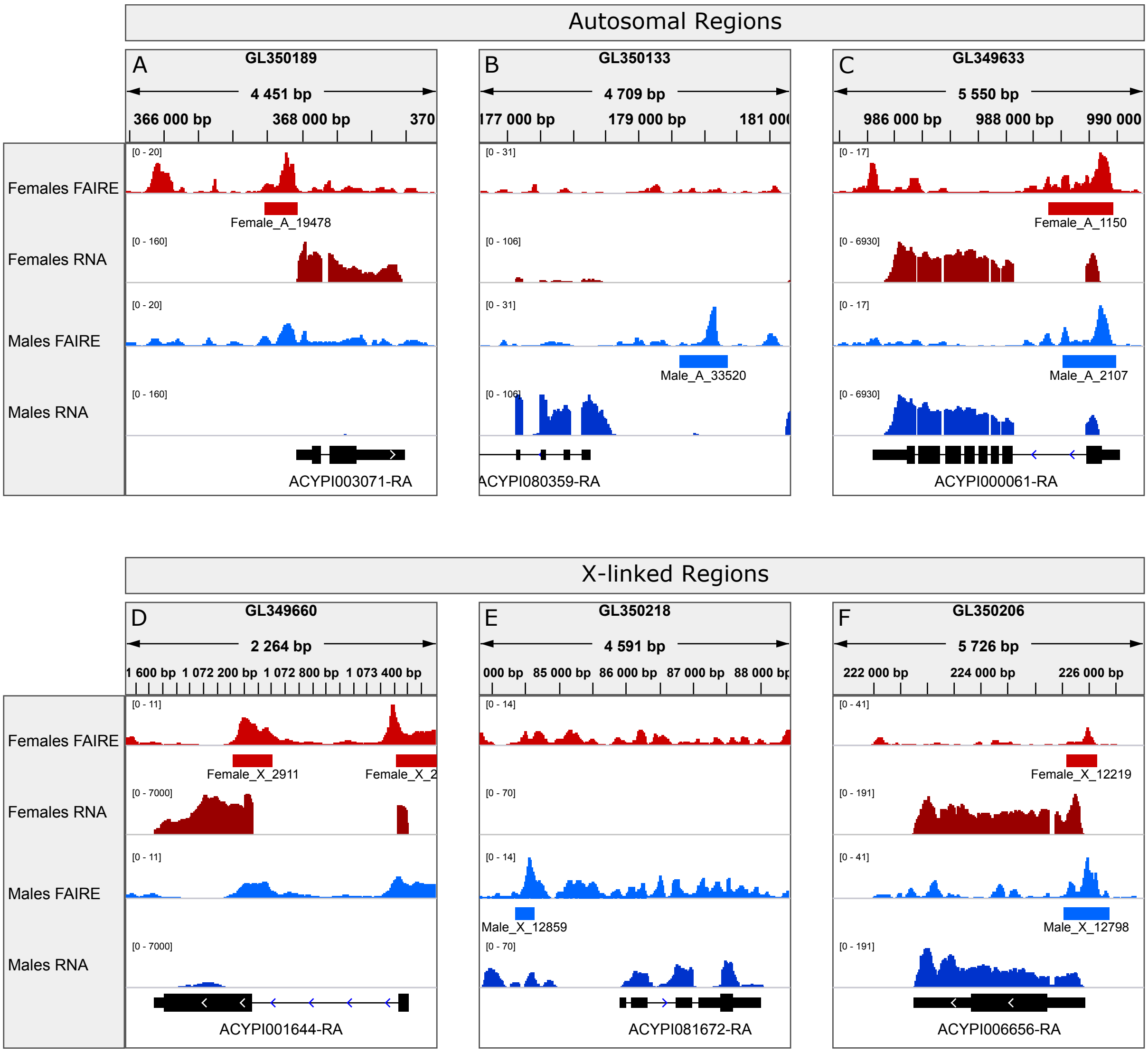

Supplement: Supplementary file 2 — Additional file 2. Genome browser view of remarkable autosomal and X-linked regions displaying sex-specific and non-specific FAIRE-seq and RNA-seq signal. A, D: female-specific regions around the genes ACYPI003071 (uncharacterized protein) and ACYPI001644 (cuticular protein 44). B, E: male-specific regions around the genes ACYPI080359 (uncharacterized protein) and ACYPI081672 (uncharacterized protein). C, F: regions in common between males and females for the genes ACYPI000061 (ATP synthase subunit beta) and ACYPI006656 (molybdate-anion transporter). The RNA-seq and FAIRE-seq signals have been made equal between males and females for each region. [file 13072_2017_137_MOESM2_ESM.pdf]
