# Supplementary material for: Dosage compensation and sex-specific epigenetic landscape of the X chromosome in the pea aphid
Source: Epigenetics Chromatin. 2017 Jun 15;10:30. doi: 10.1186/s13072-017-0137-1 (PMC5471693; doi:10.1186/s13072-017-0137-1)

Additional file 3: Mean FAIRE coverage calculated for all autosomal genes (left, black) and X-linked genes (right, grey) for males (blue) and females (red). 99% CI based on 1000 bootstrap is showed around the mean. The FAIRE coverage has been normalized by read-depth in order to allow the comparison between males and females data.

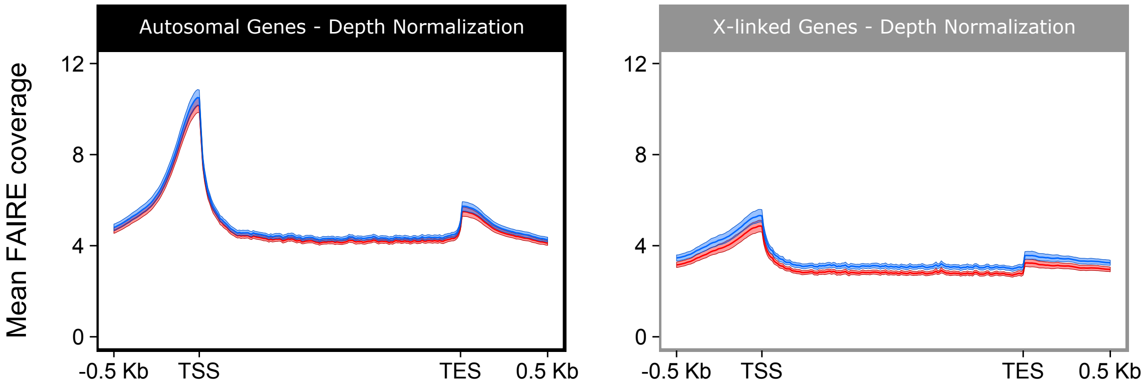

Supplement: Supplementary file 3 — Additional file 3. Mean FAIRE coverage calculated for all autosomal genes (left, black) and X-linked genes (right, gray) for males (blue) and females (red). 99% CI based on 1000 bootstrap is shown around the mean. The FAIRE coverage has been normalized by read-depth in order to allow the comparison between males and females data. [file 13072_2017_137_MOESM3_ESM.pdf]
