# Supplementary material for: Dosage compensation and sex-specific epigenetic landscape of the X chromosome in the pea aphid
Source: Epigenetics Chromatin. 2017 Jun 15;10:30. doi: 10.1186/s13072-017-0137-1 (PMC5471693; doi:10.1186/s13072-017-0137-1)

Additional file 4: Mean FAIRE signal and proportion of X-linked and autosomal genes depending on their expression profile, grouped by sex. Genes have been categorized in four different classes: *unexpressed* (**A, E**), *male-biased* (**B, F**), *female-biased* (**C, G**) and *unbiased* (**D, H**) genes between the two sexes based on gene expression data. The mean FAIRE signal has been calculated around each gene class (500bp) and in their gene body (scaled), depending on the chromosome type (autosomes in dark color, X chromosome in light color) and depending on the sex (males, top in blue and females, bottom in red. 99% CI based on bootstraps is also showed around the mean.

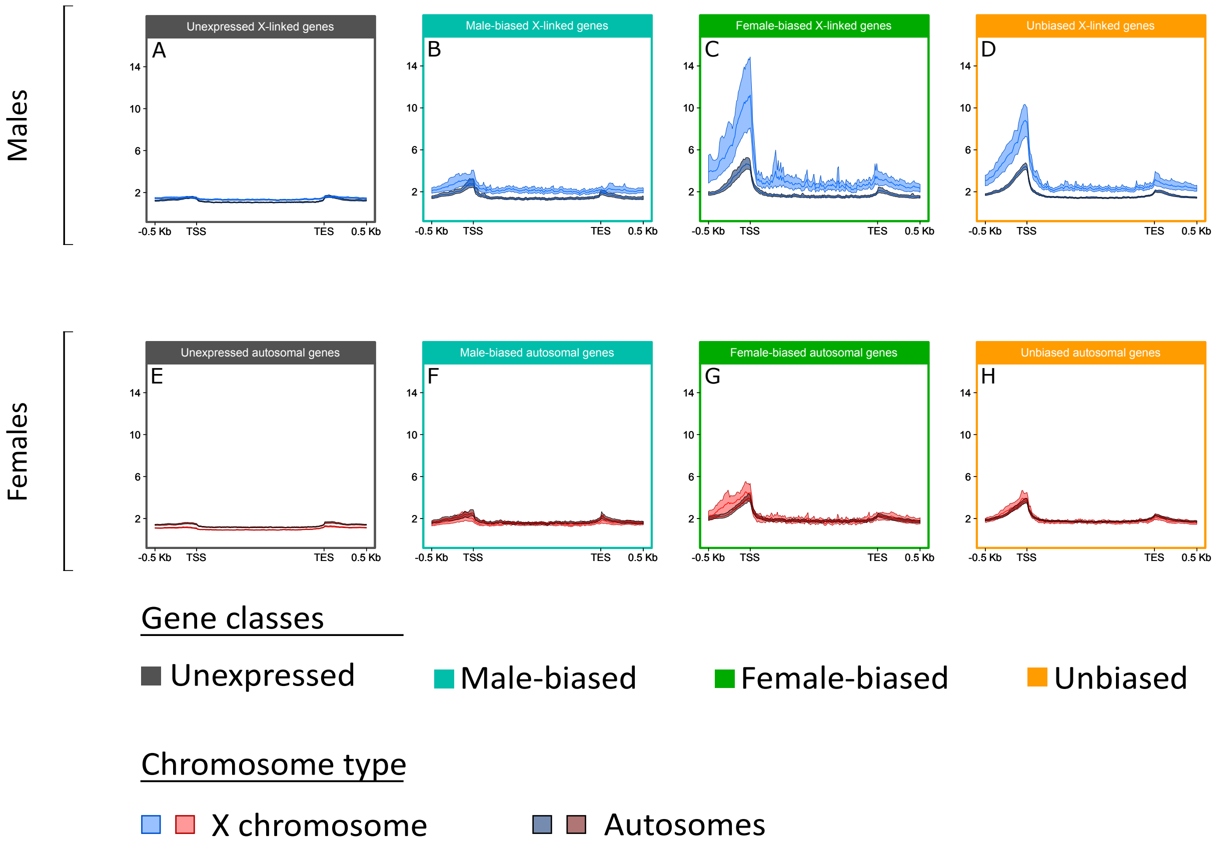

Supplement: Supplementary file 4 — Additional file 4. Mean FAIRE signal and proportion of X-linked and autosomal genes depending on their expression profile, grouped by sex. Genes have been categorized in four different classes: unexpressed (A, E), male-biased (B, F), female-biased (C, G) and unbiased (D, H) genes between the two sexes based on gene expression data. The mean FAIRE signal has been calculated around each gene class (500 bp) and in their gene body (scaled), depending on the chromosome type (autosomes in dark color, X chromosome in light color) and depending on the sex (males, top in blue and females, bottom in red. 99% CI based on bootstraps is also shown around the mean. [file 13072_2017_137_MOESM4_ESM.pdf]
