# Supplementary material for: Dosage compensation and sex-specific epigenetic landscape of the X chromosome in the pea aphid
Source: Epigenetics Chromatin. 2017 Jun 15;10:30. doi: 10.1186/s13072-017-0137-1 (PMC5471693; doi:10.1186/s13072-017-0137-1)
Supplement: Supplementary file 5 — Additional file 5. GO enrichment of biological processes for autosomal and X-linked, male- and female-biased genes. [file 13072_2017_137_MOESM5_ESM.pdf]

**Additional file 5: GO enrichment of biological processes for autosomal and X-linked, male and female-biased genes.**

**X-linked male-biased genes GO enrichment.** Enriched Biological Processes GO terms of male-biased X-linked genes with unique *D. melanogaster* homologs (n=235).

| GO term | Description | P-value | FDR q-value | Enrichment (N. B. n. b) |
|---|---|---|---|---|
| GO:0003341 | cilium movement | 1.82E-12 | 1.05E-08 | 19.59 (5349.13.210.10) |
| GO:0007018 | microtubule-based movement | 9.01E-07 | 2.60E-03 | 4.75 (5349.75.210.14) |
| GO:0007017 | microtubule-based process | 5.86E-05 | 1.13E-01 | 2.56 (5349.209.210.21) |
| GO:0006513 | protein monoubiquitination | 5.97E-05 | 8.63E-02 | 25.47 (5349.3.210.3) |
| GO:0046692 | sperm competition | 7.36E-05 | 8.52E-02 | 14.56 (5349.7.210.4) |
| GO:0044706 | multi-multicellular organism process | 1.43E-04 | 1.38E-01 | 12.74 (5349.8.210.4) |
| GO:0045814 | negative regulation of gene expression. epigenetic | 3.42E-04 | 2.83E-01 | 5.09 (5349.35.210.7) |
| GO:0040029 | regulation of gene expression. epigenetic | 6.23E-04 | 4.50E-01 | 3.40 (5349.75.210.10) |
| GO:0044703 | multi-organism reproductive process | 8.91E-04 | 5.73E-01 | 8.49 (5349.12.210.4) |

**X-linked female-biased genes GO enrichment.** Enriched Biological Processes GO terms of female-biased X-linked genes with unique *D. melanogaster* homologs (n=182).

| GO term | Description | P-value | FDR q-value | Enrichment (N. B. n. b) |
|---|---|---|---|---|
| GO:0009886 | post-embryonic animal morphogenesis | 3.97E-05 | 2.29E-01 | 4.56 (5349.309.38.10) |
| GO:0048518 | positive regulation of biological process | 4.02E-05 | 1.16E-01 | 2.85 (5349.790.38.16) |
| GO:0048583 | regulation of response to stimulus | 9.16E-05 | 1.77E-01 | 3.19 (5349.573.38.13) |
| GO:0009653 | anatomical structure morphogenesis | 1.22E-04 | 1.76E-01 | 2.75 (5349.768.38.15) |
| GO:0048522 | positive regulation of cellular process | 1.40E-04 | 1.61E-01 | 2.88 (5349.685.38.14) |
| GO:0010646 | regulation of cell communication | 1.45E-04 | 1.40E-01 | 3.28 (5349.515.38.12) |
| GO:0023051 | regulation of signaling | 1.45E-04 | 1.20E-01 | 3.28 (5349.515.38.12) |
| GO:0035120 | post-embryonic appendage morphogenesis | 2.87E-04 | 2.07E-01 | 5.27 (5349.187.38.7) |
| GO:0035114 | imaginal disc-derived appendage morphogenesis | 3.27E-04 | 2.10E-01 | 5.16 (5349.191.38.7) |
| GO:0035107 | appendage morphogenesis | 3.95E-04 | 2.28E-01 | 5.00 (5349.197.38.7) |
| GO:0002682 | regulation of immune system process | 4.00E-04 | 2.10E-01 | 6.03 (5349.140.38.6) |
| GO:0003002 | regionalization | 4.46E-04 | 2.15E-01 | 4.90 (5349.201.38.7) |
| GO:0030718 | germ-line stem cell population maintenance | 5.31E-04 | 2.36E-01 | 10.43 (5349.54.38.4) |
| GO:0009966 | regulation of signal transduction | 8.96E-04 | 3.70E-01 | 3.12 (5349.451.38.10) |

**X-linked unbiased genes GO enrichment.** Enriched Biological Processes GO terms of unbiased X-linked genes with unique *D. melanogaster* homologs (n=517).

| GO term | Description | P-value | FDR q-value | Enrichment (N. B. n. b) |
|---|---|---|---|---|
| GO:0007424 | open tracheal system development | 4.23E-04 | 1.00E+00 | 2.44 (5349.81.461.17) |
| GO:0060541 | respiratory system development | 4.23E-04 | 1.00E+00 | 2.44 (5349.81.461.17) |
| GO:0098732 | macromolecule deacylation | 8.14E-04 | 1.00E+00 | 5.80 (5349.10.461.5) |

**Autosomal male-biased genes GO enrichment.** Enriched Biological Processes GO terms of male-biased autosomal genes with unique *D. melanogaster* homologs (n=598).

| GO term | Description | P-value | FDR q-value | Enrichment (N. B. n. b) |
|---|---|---|---|---|
| GO:0006629 | lipid metabolic process | 8.50E-08 | 4.91E-04 | 2.21 (5349.214.532.47) |
| GO:0044281 | small molecule metabolic process | 1.34E-07 | 3.87E-04 | 1.85 (5349.374.532.69) |
| GO:0044710 | single-organism metabolic process | 1.83E-07 | 3.52E-04 | 1.54 (5349.776.532.119) |
| GO:0055085 | transmembrane transport | 7.74E-07 | 1.12E-03 | 2.14 (5349.202.532.43) |
| GO:0044712 | single-organism catabolic process | 2.96E-05 | 3.42E-02 | 2.41 (5349.100.532.24) |
| GO:0042445 | hormone metabolic process | 4.14E-05 | 3.99E-02 | 3.66 (5349.33.532.12) |
| GO:0055114 | oxidation-reduction process | 4.20E-05 | 3.47E-02 | 1.86 (5349.227.532.42) |
| GO:0006091 | generation of precursor metabolites and energy | 1.20E-04 | 8.67E-02 | 2.99 (5349.47.532.14) |
| GO:0006820 | anion transport | 1.49E-04 | 9.58E-02 | 3.26 (5349.37.532.12) |
| GO:0015698 | inorganic anion transport | 2.03E-04 | 1.17E-01 | 3.93 (5349.23.532.9) |
| GO:0044255 | cellular lipid metabolic process | 2.36E-04 | 1.24E-01 | 1.95 (5349.155.532.30) |
| GO:0006082 | organic acid metabolic process | 3.93E-04 | 1.90E-01 | 1.77 (5349.204.532.36) |
| GO:0044262 | cellular carbohydrate metabolic process | 4.15E-04 | 1.85E-01 | 3.35 (5349.30.532.10) |
| GO:0006865 | amino acid transport | 4.22E-04 | 1.74E-01 | 3.62 (5349.25.532.9) |
| GO:0030239 | myofibril assembly | 5.15E-04 | 1.99E-01 | 5.03 (5349.12.532.6) |
| GO:0006099 | tricarboxylic acid cycle | 5.72E-04 | 2.07E-01 | 3.83 (5349.21.532.8) |
| GO:0044282 | small molecule catabolic process | 5.92E-04 | 2.01E-01 | 2.61 (5349.54.532.14) |
| GO:0015849 | organic acid transport | 8.08E-04 | 2.60E-01 | 3.35 (5349.27.532.9) |
| GO:0006979 | response to oxidative stress | 8.41E-04 | 2.56E-01 | 2.43 (5349.62.532.15) |
| GO:0032787 | monocarboxylic acid metabolic process | 8.64E-04 | 2.50E-01 | 2.28 (5349.75.532.17) |
| GO:0043648 | dicarboxylic acid metabolic process | 8.77E-04 | 2.41E-01 | 4.64 (5349.13.532.6) |
| GO:0006121 | mitochondrial electron transport. succinate to ubiquinone | 9.79E-04 | 2.57E-01 | 10.05 (5349.3.532.3) |

**Autosomal female-biased genes GO enrichment.** Enriched Biological Processes GO terms of female-biased autosomal genes with unique *D. melanogaster* homologs (n=1107).

| GO term | Description | P-value | FDR q-value | Enrichment (N. B. n. b) |
|---|---|---|---|---|
| GO:0002181 | cytoplasmic translation | 2.75E-32 | 1.59E-28 | 4.31 (5349.74.1023.61) |
| GO:0006807 | nitrogen compound metabolic process | 1.69E-20 | 4.89E-17 | 1.52 (5349.1144.1023.332) |
| GO:0034641 | cellular nitrogen compound metabolic process | 1.80E-20 | 3.46E-17 | 1.56 (5349.1020.1023.304) |
| GO:0022402 | cell cycle process | 4.88E-17 | 7.05E-14 | 1.89 (5349.401.1023.145) |
| GO:0090304 | nucleic acid metabolic process | 1.85E-15 | 2.14E-12 | 1.62 (5349.672.1023.208) |
| GO:0051298 | centrosome duplication | 2.61E-13 | 2.51E-10 | 3.56 (5349.47.1023.32) |
| GO:0009059 | macromolecule biosynthetic process | 1.59E-12 | 1.32E-09 | 1.74 (5349.399.1023.133) |
| GO:0031023 | microtubule organizing center organization | 2.18E-12 | 1.58E-09 | 2.73 (5349.88.1023.46) |
| GO:0034645 | cellular macromolecule biosynthetic process | 2.69E-12 | 1.73E-09 | 1.79 (5349.354.1023.121) |
| GO:0006139 | nucleobase-containing compound metabolic process | 4.14E-12 | 2.39E-09 | 1.48 (5349.789.1023.224) |
| GO:1901360 | organic cyclic compound metabolic process | 4.43E-12 | 2.33E-09 | 1.45 (5349.883.1023.245) |
| GO:0006412 | translation | 7.71E-12 | 3.71E-09 | 2.16 (5349.172.1023.71) |
| GO:0051297 | centrosome organization | 1.03E-11 | 4.58E-09 | 2.71 (5349.85.1023.44) |
| GO:0006396 | RNA processing | 1.16E-11 | 4.79E-09 | 1.83 (5349.306.1023.107) |
| GO:0043043 | peptide biosynthetic process | 1.26E-11 | 4.87E-09 | 2.13 (5349.177.1023.72) |
| GO:0032502 | developmental process | 1.28E-11 | 4.61E-09 | 1.27 (5349.1787.1023.434) |
| GO:0016071 | mRNA metabolic process | 1.30E-11 | 4.42E-09 | 1.97 (5349.231.1023.87) |
| GO:1903047 | mitotic cell cycle process | 1.38E-11 | 4.44E-09 | 1.90 (5349.261.1023.95) |
| GO:0006725 | cellular aromatic compound metabolic process | 1.47E-11 | 4.46E-09 | 1.45 (5349.856.1023.237) |
| GO:0016070 | RNA metabolic process | 1.68E-11 | 4.87E-09 | 1.60 (5349.522.1023.160) |
| GO:0046483 | heterocycle metabolic process | 2.30E-11 | 6.32E-09 | 1.45 (5349.828.1023.230) |
| GO:0043604 | amide biosynthetic process | 2.39E-11 | 6.28E-09 | 2.10 (5349.179.1023.72) |

**Autosomal unbiased genes GO enrichment.** Enriched Biological Processes GO terms of unbiased autosomal genes with unique *D. melanogaster* homologs (n=2805).

| GO term | Description | P-value | FDR q-value | Enrichment (N. B. n. b) |
|---|---|---|---|---|
| GO:0051179 | localization | 7.61E-07 | 4.40E-03 | 1.14 (5349.1063.2508.569) |
| GO:0006810 | transport | 1.66E-06 | 4.80E-03 | 1.15 (5349.910.2508.491) |
| GO:0051234 | establishment of localization | 7.24E-06 | 1.40E-02 | 1.14 (5349.945.2508.504) |
| GO:0006898 | receptor-mediated endocytosis | 1.94E-05 | 2.80E-02 | 1.73 (5349.37.2508.30) |
| GO:0007264 | small GTPase mediated signal transduction | 6.54E-05 | 7.57E-02 | 1.44 (5349.89.2508.60) |
| GO:0072583 | clathrin-dependent endocytosis | 6.71E-05 | 6.47E-02 | 1.73 (5349.32.2508.26) |
| GO:0035556 | intracellular signal transduction | 1.08E-04 | 8.90E-02 | 1.26 (5349.221.2508.131) |
| GO:0016192 | vesicle-mediated transport | 1.39E-04 | 1.01E-01 | 1.20 (5349.355.2508.200) |
| GO:0048488 | synaptic vesicle endocytosis | 2.05E-04 | 1.32E-01 | 1.71 (5349.30.2508.24) |
| GO:0099643 | signal release from synapse | 3.78E-04 | 2.19E-01 | 1.40 (5349.82.2508.54) |
| GO:0001505 | regulation of neurotransmitter levels | 3.85E-04 | 2.02E-01 | 1.38 (5349.91.2508.59) |
| GO:0023061 | signal release | 4.17E-04 | 2.01E-01 | 1.40 (5349.84.2508.55) |
| GO:0099003 | vesicle-mediated transport in synapse | 4.35E-04 | 1.93E-01 | 1.55 (5349.44.2508.32) |
| GO:0006464 | cellular protein modification process | 5.41E-04 | 2.24E-01 | 1.14 (5349.601.2508.320) |
| GO:0036211 | protein modification process | 5.41E-04 | 2.09E-01 | 1.14 (5349.601.2508.320) |
| GO:0099531 | presynaptic process involved in chemical synaptic transmission | 6.48E-04 | 2.34E-01 | 1.38 (5349.85.2508.55) |
| GO:0070647 | protein modification by small protein conjugation or removal | 7.97E-04 | 2.71E-01 | 1.29 (5349.139.2508.84) |
| GO:0007269 | neurotransmitter secretion | 9.15E-04 | 2.94E-01 | 1.39 (5349.75.2508.49) |
