# Supplementary material for: Dosage compensation and sex-specific epigenetic landscape of the X chromosome in the pea aphid
Source: Epigenetics Chromatin. 2017 Jun 15;10:30. doi: 10.1186/s13072-017-0137-1 (PMC5471693; doi:10.1186/s13072-017-0137-1)
Supplement: Supplementary file 6 — Additional file 6. Protein sequence comparison of the Drosophila’s DCC in the pea aphid. Clustal multiple sequence alignment by MUSCLE (3.8) of A. pisum’s homologs of D. melanogaster DCC. Proteic domains of the Drosophila are represented in full color boxes above the alignments. A. pisum gene names found by BLASTp are indicated in parenthesis. [file 13072_2017_137_MOESM6_ESM.pdf]

Additional file 6: Protein sequence comparison of the Drosophila's DCC in the pea aphid. CLUSTAL multiple sequence alignment by MUSCLE (3.8) of *A. pisum*'s homologs of *D. melanogaster* DCC. Proteic domains of the Drosophila are represented in full color boxes above the alignments. *A. pisum* gene names found by BLASTp are indicated in parenthesis.

MSL1 (XM_003243415.1)

```
MSL1_DROME      MDKRFKWPPKKRANYLESPYPHIPSRGRQRNLHGHPNQTQHLHQHPGKIYERQQYGNGRG
MSL1_ACYPI      MARR-----------------------------------------------RNSRGVGRA
                * .*                                               *:. * **.

MSL1_DROME      GHGGGNNNYRKLLHSLPAEHGGGAMAPPSSGGTVCAGADMVKLISENNNLRRMVMLNLNL
MSL1_ACYPI      ------------------------------------------------------------

MSL1_DROME      MQEQTDSIAAKDKELDDQSAKMSVVKAQNEELKQAVAQLEAANQELCKQLRRKNQRRNDN
MSL1_ACYPI      --------SAMSKEKDEK------------------------------------------
                        :* .** *::

MSL1_DROME      DDDDDDPPLPPAAPQQKLIRCHAETQTVFREREQGTQTIDAQPQFANALPRGINMKESPA
MSL1_ACYPI      ------------------------------------------------------------

MSL1_DROME      LDHHAGAVTNQPASKRSESKGRGEFNGKKVSTFILQRMNQDFEHHIHEQTEVAEEHEMEA
MSL1_ACYPI      ---------------------------------IL-------------------------
                                                 **

MSL1_DROME      HKEQISQEEDQLVAEEDHLHMQEVHTEEVVGGDIFHDALESIEMEVVTEELVDMEEHGQS
MSL1_ACYPI      ---QLTNEVEYLKQKSDVLSFTQVSS----------------------------------
                   *:::* : *  :.* * : :* :

MSL1_DROME      VDANGHIEEDDDEDDEDDENSDKDDDSEEDDYPWMHSDADVNARTEEELWQNQNYLLELD
MSL1_ACYPI      ------------------------------------------------------------

MSL1_DROME      PTEEKTCAPSAHSTPNHQQKSSTQAEIRKEGNQNRITEKLLQLKPEPMVDALEAPILPKW
MSL1_ACYPI      -----TCSS---------------------------------------------------
                     **:.

MSL1_DROME      VAFKKKDKEHESVPESPEVPKQQPHQEDAIVDHNAIKNQLEVPKPDLKPKDQPKDEQRQD
MSL1_ACYPI      ----------------------------GIGDLSLAEKQYQQEHQHIKPK----------
                                            .* * .  ::* :  :  :***

MSL1_DROME      GQLDVRVEPQEDVRKVQKETLKRQPEDAPKHLPKAVAPKVTKTSSRESTLPKANTADIKD
MSL1_ACYPI      -------------KRTIRQAATKQPNPDP--------LESNQTKRRRTSIPQTKSAKLKN
                             ... .:: ..**:  *         : .:*. * :::*::::*.:*:

MSL1_DROME      APAQKVIANHQSTKT---QTDPVKTQRLQVKIRQYEMHPDMRTGSSAPSDIRKQKNVDPV
MSL1_ACYPI      TEELSLITADDAYYTCLGSYDPIKS--------------------------------NPV
                :   .:*:  ::  *   . **:*:                                :**

MSL1_DROME      STPETKTIKSKSMLVNDKKTTSETSQSPDQEIDVETVRRKLAEHLKKELLSQSHSSQVTL
MSL1_ACYPI      SKNTKRNI----------------------------------------------------
                *.  ...*

MSL1_DROME      KKIRERVATNLIYPPPSAPVSSTTITPAPTPSTTPTPGSTPQHAVTSSMDQEISAAKSKS
MSL1_ACYPI      ------------------------------------------------------------

MSL1_DROME      KAAEQIATPLTPQSNSSVSSTTSTIRKTLNNCSPHTYSKATARSGKLQSRFRTATFPYST
MSL1_ACYPI      ------------------------------------------------------------
```

```
MSL1_DROME      RTWEDQEFHCDNEFFLEEADELLADNPSLEIPKWRDVPVPPSSDKIDTELLSDATFERRH
MSL1_ACYPI      -----------------------------EIPNYRLKKYSCRNSAKGIELMSDAIFQKRH
                                             ***::*    .  ..  . **:*** *:.**

                                             PEHE
MSL1_DROME      QKYVKDEVDRKCRDARYMKEQIRLEQLRMRRNQDEVLVALDPLRA--STFYPLPEDIEAI
MSL1_ACYPI      FKHERSEQICKRRDLRQIRELIRVENLRKGRNKHHSQKSNDNNKEELTTLLPSPSNIKSI
                 *: ..*   * ** * :.* **:*:**  **:     : *  .   :*: * *.:*::*

MSL1_DROME      QFVNEVTVQAFGENVVNMEARDDFGVPWVDAIEAPTSIARSKALAEPVATLASKKIPTTA
MSL1_ACYPI      EITNKLPVSAFGINLYRLTC----------------------------------------
                ::.*::.*.*** *: .: .

MSL1_DROME      AEARHQENHSSYVFPKRRKRQKNR
MSL1_ACYPI      ------------------------
```

MSL2 (ACYPI49193)

```
MSL2_DROME      MAQTAYLKVTRIAMRSASNLSKRRVEELNSGLGELRQLLSCVVCCQLL--VDPYSPKGKR
MSL2_ACYPI      MALIEYYLSTSQIVLQAKSTDPLTWQKLHSLLPLLHEHLSCQVCHKLVDRLNPY------
                **   *   *   : .*.. .    ::*:* *  *.: *** ** :*:  ::**

                                zf-RING10
MSL2_DROME      CQHNVCRLCLRGKKHLFPSCTQCEGCSDFKTYEENRMMAAQLLCYKTLCVHLLHSALFGE
MSL2_ACYPI      LDGYACTVCVNHQITENPS-------------------SAIIQCYKKLCAYIHSSPLYKV
                 :  .* :*:. :    **                   :* : ***.**.::  *.*:

MSL2_DROME      LAGMRPQVARELVPRIKLPPKTTQEFIREGSNISDTFDIFLPQPDLPFLKDMPTSLPAET
MSL2_ACYPI      MC-------------TRVEDKRLVELVTE-------------------------------
                :.             .:  *   *:: *

MSL2_DROME      PPTSAVTTPELPYDHHLNISDIEAEAAATAEQGHFSPLPLLPTGSRMGMLSHAGQIVIAT
MSL2_ACYPI      --------------------------------------------------------VISL
                                                                        **:

MSL2_DROME      ESSESGFMDQAWTDQVDLSGTVSVSKYTNSGNNFAVSYVMPTSATTKFDPQELQIGQVVQ
MSL2_ACYPI      PRSINGY-----------SGMVN---------------------------------GTVIK
                  * .*:           ** *.                                 * *::

MSL2_DROME      MADSTQLAVLAAVEETVETSTQLTVLSTTVEETVETSTQLEVLTSAEEPNEISDQLANLQ
MSL2_ACYPI      QEKK---------EKDIEDDSTLMVNGTMNKQEIK-------------------------
                  ..         *: :* .: * * .*  :: ::

MSL2_DROME      VEESDEALVEETVEEAEGTSIPSEVVAEHMEEDQHLDVHTSQSPTQTEMEEAVEEHVATK
MSL2_ACYPI      ----EEDIEDDSTLMVNGTMNKQEIKEEDIEDDFTLMVNGIMNKQEIKEENSKDDI----
                    :* : :::.  .:**   .*:  * :*:*  * *:    .  : : *:: ::

MSL2_DROME      TQLGHVQTELQDAESLQKDFEDAKAAAEEAKEKEKDLHAISAELQKEDSDEPTLKRKRTR
MSL2_ACYPI      TLISSSDIQLPTVNETPEDYINYQIFDVQSQ-----------------------------
                * :.  : :*  .:.  :*: : :    :::

MSL2_DROME      TLKASQAAKIEPVPSEVKTKVQSGKGALRRIRGKDKEEKVKPPKPKCRCGISGSSNTLTT
MSL2_ACYPI      -----------------KHKIQN-QSVFNNIPKKKKNER----RWGCRCGNATTTPGKLT
                                 * *:*. :..:..*  *.*:*.    .  **** : ::    *

                  MSL2-CXC
MSL2_DROME      CRNSRCPCYKSYNSCAGCHCVCCKNPHKEDYVESDEDDDLEDFEMPKDVPEPMTQSEEPV
MSL2_ACYPI      CFGQRCPCYTEQKPCDQCKCRGCRNPRQK---RSNNDDNLE-------------------
                * ..*****.. :.*  *:*  *.**.::    *::**:**
```

```
MSL2_DROME      VAEPRQEENSMAPPDSSAPISLVPLNNLQQSQHPLVLVQNEKGEYQGFNIFQGSKPLDPV
MSL2_ACYPI      IDQIRRK-----------PVTLELVSSLKPSSH-----NNSNSLFSAYTMHD--------
                : : *.:           *::*  :..*: *.*     :*.:. :..:.:.:

MSL2_DROME      TVGFTIRVQLQHTDGFGSLPQYAYIMPTIDPPNPPAPSLSPPPPPAPDREVIEPPAKKFR
MSL2_ACYPI      MLQFS-----SHSQQFD-------------------------------------------
                 : *:     .*:: *.

MSL2_DROME      TSRTRRGRANFSALDTVDELVSGGSRSNSAAGDRSSATDNAHSLFEEIMSGSDDL
MSL2_ACYPI      ---------------------QGGTEDN---------LGHVNSIFHSP-------
                                     .**: .*           .:.:*:* .
```

MSL3 (ACYPI000966)

```
MSL3_DROME      MTELRDET-PLFHKGEIVLCYEPDKSKARVLYTSKVLNVFERRNEHGLRFYEYKIHFQGW
MSL3_ACYPI      MAAKKVLTKSKFEEGEKVLCYEPDPAKTKVLYDSKVLRVVPEKDEQGRKFCKFLIHFQGW
                *:  .  * . * :** ******* :*:.*** ****.*.  .:*:* .* :: ******

MSL3_DROME      RPSYDRCVRATVLLKDTEENRQLQRELAEAAKLQIRGDYSYKGTPDKPSAKKKRGGKAAH
MSL3_ACYPI      NSTWDRFVTDEFILKDTEENRKLQKELAEEAQLTPGGNL-YRKERKKRAVKLE---PKPL
                ..::** *   .:********:**.**** *:*   *:  *.   .* :.* :     .

MSL3_DROME      VEEPIVVPMDTGHLEAEHEMAPTPRAAGNRTRDNSGGKRKEKPPSGDGRLKGNRGRQTET
MSL3_ACYPI      VIEPAVVTIDDKSM-VENENVPVP------------------------------------
                * ** **.:*   : .*:* .*.*

MSL3_DROME      FYNNAINDVSVYNHVPQEDRIMMRVSERLRELIEYDRNMIKVLGKQHALPARVPIVTIME
MSL3_ACYPI      -----IDTI----LLPKRRLPDLEFPDNLKFHTGYNCYLVHEKNTLVQLPCQPNVVTLLE
                     *: :     :*:     : ..:.*.    *:  :::  ..   **..  :**::*

MSL3_DROME      NFVKQQAVELAISIKQDSSRARNTQSRNARMEREYDRVMSTVCMLKEVVDGLRIYFEFHV
MSL3_ACYPI      SYLRYLARNNFSDNKTTKKKRQPEVLDKKQLEKRY-----IICV--EVLDGLRICFNTFL
                .::.  * :   . *  ... .     : .:*. *      :*:  **:***** *: .:

                                            MRG
MSL3_DROME      DDHLLYTEEKE---YVHNYLTDDNMRNCSLILNKSYEYINPSGDTELIGLDGTPVVEGSG
MSL3_ACYPI      FRKLLVNEDEQAQYYEALKVTLQPPVNNIIPQNGEQDYDNVPNDEESESHDNKNNVKAKR
                  :** .*:::   *    :* :   *  :  * . :* * ..* *  . *..  *:..

MSL3_DROME      DTNGQIGVINIGGPEYEKQLQKCLLYIVTAS--GKNTAQAYERTSPYTAAYKLPVEMRGF
MSL3_ACYPI      DNTRS------STSQYASSQQTCSDCSLTKSQCSKIVKDTFEKARD------------EY
                *.. .      . .:* .. *.*    :* *  .* . :::*.:               :

MSL3_DROME      LNETFKWRLLS----AESPPEKSMVFGAPHLVRLMIKMPMFLNASPISNKKLEDLLPHLD
MSL3_ACYPI      VSKADSWKAVPDSAYDEEIKQPAVVYGVYHLLRLLENLPKILANTEVDGEKLSIVYLYSN
                :.:: .*. :.     *.  : ::*:*. **:**: ::* :*  : :..:**. :  : :

MSL3_DROME      AFINYLENHREWFDRENFVNSTALPQEDLQRELLDSLDGIAA
MSL3_ACYPI      GLLKYLSTQTYLFGMQYYVKNEMEDAVKSSAPIRNHRNHKNI
                .:::**..:   *. : :*:.      . .  : :  :
```

MOF (ACYPI002102)

```
MOF_DROME       MSEAELEQTPSAGHVQEQPIEEEHEPEQEPTDAYTIGGPPRTPVEDAAAELSASLDVSGS
MOF_ACYPI       -----------------------------------------MVVHHSATSVSMEVD----
                                                           *  :*:.:* .:*

MOF_DROME       DQSAEQSLDLSGVQAEAAAESEPPAKRQHRDISPISEDSTPASSTSTSSTRSSSSSRYDD
MOF_ACYPI       ------------------------------------------------------------

MOF_DROME       VSEAEEAPPEPEPEQPQQQQQEEKKEDGQDQVKSPGPVELEAQEPAQPQKQKEVVDQEIE
MOF_ACYPI       ------------------------------------------------------------

MOF_DROME       TEDEPSSDTVICVADINPYGSGSNIDDFVMDPDAPPNAIITEVVTIPAPLHLKGTQQLGL
MOF_ACYPI       ------------------------------------------------------------

MOF_DROME       PLAAPPPPPPPPAAEQVPETPASPTDDGEEPPAVYLSPYIRSRYMQESTPGLPTRLAPRD
MOF_ACYPI       ----------PPRNANSPDDPKT-------------------------------------
                          **   : *: * :

MOF_DROME       PRQRNMPPPAVVLPIQTVLSANVEAISDDSSETSSSDDDEEEEEDEDDALTMEHDNTSRE
MOF_ACYPI       --------------------------ANGSSENEKAEDSESSEDG---------------
                                          ::.***...::*.*..*:.

                                                   Tudor-knot
MOF_DROME       TVITTGDPLMQKIDISENPDKIYFIRREDGTVHRGQVLQSRTTENAAAPDEYYVHYVGLN
MOF_ACYPI       -----------PLDVGEH----YLVRRYDDSWHPAEVLHTRFNDTEHLF-EYYVHYEGYN
                            :*:.*:    *::** *.: * .:**::* .:.     ****** * *

MOF_DROME       RRLDGWVGRHRISDNADDLGGITVLPAPPLAPDQPSTSREMLAQQAAAAAAASSERQKRA
MOF_ACYPI       RRLDQWVPRDRIMNSRFDI--------------------------------SEKNWKQKD
                **** ** * ** :.  *:                                :..:.::.

MOF_DROME       ANKDYYLSYCENSRYDYSDRKMTRYQKRRYDEINHVQKSHAELTATQAALEKEHESITKI
MOF_ACYPI       VNKNLDL-------LNQSDRKITRNQKRRHDEINHVPMSYAEMDPTTAALEKEHEAITKI
                .**:  *        : ****:** ****:******  *:**: .* ********:****

MOF_DROME       KYIDKLQFGNYEIDTWYFSPFPEEYGKARTLYVCEYCLKYMRFRSSYAYHLHECDRRRPP
MOF_ACYPI       KYINKVQLGKYEIDTWYFSPFPAEYQKESKIWICEYCLKYSKLEKSFKYHMSQCTWRQPP
                ***:*:*:*:************ ** *  .:::******* .: .*: **: :* .*.**

MOF_DROME       GREIYRKGNISIYEVNGKEESLYCQLLCLMAKLFLDHKVLYFDMDPFLFYILCETDKEGS
MOF_ACYPI       GVEVYHKGSLSIWEVNSSQHKMYCQNLCLLAKLFLDHKTLYFDVEPFLFYILCEVDKVGA
                * *:*.**.:**:***..: .:*** ***:********.****::*********.** *:

                                          MOZ-SAS
MOF_DROME       HIVGYFSKEKKSLENYNVACILVLPPHQRKGFGKLLIAFSYELSRKEGVIGSPEKPLSDL
MOF_ACYPI       HLVGYFSKEKDSPDCNNVACILTMPPFQRQGYGKLLIAFSYELSKLEGLVASPEKPLSDL
                *:********.* :  ******.:**.**:*:************. **::.*********

MOF_DROME       GRLSYRSYWAYTLLELMKTRCAPEQITIKELSEMSGITHDDIIYTLQSMKMIKYWKGQNV
MOF_ACYPI       GKLSYRSYWSWVLLEILKNACGS--LSIKDLSAMTSITQTDIISTLQSMNMVKYWKGQHV
                *.*******::.***::*. *..  ::**:** *:.**: *** *****:*:******:*

MOF_DROME       ICVTSKTIQDHLQLPQFKQPKLTIDTDYLVWSPQT------AAAVVRAPGNSG
MOF_ACYPI       ICVTPKNVEQLVSSEQYKRPRFILEGSAIKWTPKKYNPRPGPSRHSRSQYLAG
                ****.*.::: :.  *:*.*.: :: . : *:*:.      .:   *:   :*
```

MLE (ACYPI003650)

```
MLE_DROME       ------------------------------------------------------------
MLE_ACYPI       MANPKAYLHEWCAKNNLELQFETKQAGSNCKPRLFICEVTVTGHNYIGVGNSTNKKLAQA

MLE_DROME       ------------------------------------------------------------
MLE_ACYPI       NASKDYLLYLTKEGLISIDTYFLPSNCISGPASNGLMVNASQQLMNNVPPKSEYEEETSN

MLE_DROME       ------------------------------------------------------------
MLE_ACYPI       ILGEAYRPNEKTNFQLNDFLNDENNVEEAEGLNLHAAVCGDWTIDNAKSHLNQFIQSNKL

                                             dsrm
MLE_DROME       --------MDIKSFLYQFCAKSQIEPKFDIRQTGPKNRQRFLCEVRVEPNTYIGVGNSTN
MLE_ACYPI       KNIDYKYSCNPKTFLHEWCEKNNLEPQFKTKQAGSNRKPRFICEVIITGHDYIGIGNSAN
                         : *:**:::* *.::**:*. .*:*.:.. **:*** :  : ***:***:*

MLE_DROME       KKDAEKNACRDFVNYLVRVGKLNTNDVPADAGASGGGPRTGLEGAGMAGGSGQQKRVFDG
MLE_ACYPI       IKYAQENASKDFLLYLTRKGLVSTDSLASNC-ISGPASNDLMVNASQKLMNVPPKSVYQD
                 * *::**..**: **.* * :.*:.:.::.  ** ...  : .*.    .   * *::.

MLE_DROME       QSGPQDLGEAYRPLNHDGGDGGNRYSVIDRIQEQRDMNEAEAFDVNAAIHGNWTIENAKE
MLE_ACYPI       HETPKMLGESYRPV------GKANFQFNDFLNDENNVEEAEHLDVNAAVHGGWTIDNAKS
                :. *: ***:***:      *  .:.. * ::::.:::*** :*****:**.***:***.

MLE_DROME       RLNIYKQTNNIRD-DYKYTPVGPEHARSFLAELSIYVPALNRTVTARESGSNKKSASKSC
MLE_ACYPI       RLNQFIQSNKLKNTDYKYSFIG----KSHVAELSIYIAKLGRNVTARESGLNKRSASTSC
                *** : *:*::.: ****: :*    .*.:******:. *.*.******* **.***.**

MLE_DROME       ALSLVRQLFHLNVIEPFSGTLKKKKDEQLKPYPVKLSPNLINKIDEVIKGLDLPVVNPRN
MLE_ACYPI       ALSFIRQLYHLGVIEPFSGSLKKSILNTVKPYEVNVDPLILNKVYNILQEFNIEPVIKND
                ***::***:**.*******:***.  : :*** *::.* ::**: :::: :::  *  .:

MLE_DROME       IKIELDGPPIPLIVNLSRIDSSQQDGEKRQESSVIPWAPPQANWNTWHACNIDEGELATT
MLE_ACYPI       TIIEPE-TTISLISNQKLAEFKELKPIKL--SRAILWSPPQPNWNPWLAINIDEGPLATA
                  ** : ..*.** * .  : .: .  *   * .* *:***.***.* * ***** ***:

MLE_DROME       SIDDLSMDYERSLRDRRQNDNEYRQFLEFREKLPIAAMRSEILTAINDNPVVIIRGNTGC
MLE_ACYPI       TLDQLSEELAKDYTKKLNSSKGFKESIEMRSQLPVFNKKNEILDIIRKNSIVIIQGSTGC
                ::*:** :  ..  .. :..: :.: :*:*.:**:    ..***  *..*.:***.*.***

                                          DEAD
MLE_DROME       GKTTQIAQYILDDYICSGQGGYANIYVTQPRRISAISVAERVARERCEQLGDTVGYSVRF
MLE_ACYPI       GKTTQVCQFILDEYLKNDQGAYCNIICTQPRKISAISIADRVAFERKEDIGLSVGYSVRF
                *****:.*:***:*: ..**.*.**  ****.*****:*:*** ** *::* :*******

MLE_DROME       ESVFPRPYGAILFCTVGVLLRKLEAGLRGVSHIIVDEIHERDVNSDFLLVILRDMVDTYP
MLE_ACYPI       DSIFPRSTGAILFCTVGVLLRKLENGMRGISHVIVDEIHERGVNSDFLMVVLKDMVYNYP
                :*:***. *************** *:**:**:********.******:*:*.***  .**

MLE_DROME       DLHVILMSATIDTTKFSKYFGICPVLEVPGRAFPVQQFFLEDIIQMTDFVPSAESRRKRK
MLE_ACYPI       DLRVIFMSATINTAMFSKYFNCCPVIDIKGRCYPVKEYFLEDIVQVLNYQPTPDIKKRMN
                **.**:*****:*: *****. ***::: **.:**:::*****:*: :: *:.: ... :

MLE_DROME       EVEDEEQLLSEDKDEAEINYNKVCEDKYSQKTRNAMAMLSESDVSFELLEALLMHIKSK-
MLE_ACYPI       KDYDDEYVI--DAQDHEENCNLLVSDDYPPEVKSKVALISEKYVDFEIIEALLTHIEIKM
                :  *:* ::  * :: * * * : .*.*. :... :*::**. *.**::**** **: *
```

```
                             Helicase C
MLE_DROME       NIPGAILVFLPGWNLIFALMKFLQNTNIFGDTSQYQILPCHSQIPRDEQRKVFEPVPEGV
MLE_ACYPI       NIPGAVLIFLPGWTLISALQKYLTEKQFFA-SSKFCVLPLHSQLPCADQRRVFEPVPSGV
                *****:*:*****.** ** *:* :.::*. :*:: :** ***:*  :**.******.**

MLE_DROME       TKIILSTNIAETSITIDDIVFVIDICKARMKLFTSHNNLTSYATVWASKTNLEQRKGRAG
MLE_ACYPI       RKVILSTNIAETSITIDDVVFVINYGKAKIKFFTSHNNMTHYATVWASKTNMQQRKGRAG
                 *:***************:****:  **.:*:******:* **********::*******

MLE_DROME       RVRPGFCFTLCSRARFQALEDNLTPEMFRTPLHEMALTIKLLRLGSIHHFLSKALEPPPV
MLE_ACYPI       RVSDGFCFHLCTKARYDKMDDHVTPEMFRSPLHEIALSIKLLRLGDIGQFLSKAIEPPPI
                **  **** **:.**:: ::*::******:****:**:*******.* :*****:****:

MLE_DROME       DAVIEAEVLLREMRCLDANDELTPLGRLLARLPIEPRLGKMMVLGAVFGCADLMAIMASY
MLE_ACYPI       DAVNEALVMLKEMKCLGINEELTPLGRILAKLPVEPQIGRMMVLGNILMLGDAFAIIAAI
                *** ** *:*.**.**. *:*******:**.**:**.:*.***** ::  .* :**:*:

                                HA2
MLE_DROME       SSTFSEVFSLDIGQRRLANHQKALSGTKCSDHVAMIVASQMWRREKQRGEHMEARFCDWK
MLE_ACYPI       CSNMTDIFVFD---HRMTPAQRAFSGNRCSDHLALLNAFQQW-QSLDYCNINPTEYCERK
                .*.::::* :*   .*::  *.*:**..****:*:: * * * .. :  :   : :*:.*

MLE_DROME       GLQMSTMNVIWDAKQQLLDLLQQAGFPEECMISHEVDERID-GDDPVLDVSLALLCLGLY
MLE_ACYPI       MLSEPSLTTTADAMEQLKDLFIKIGFPEICFEKQRFDFGVEMHDDPLLDVVSAILTMGFY
                 *. .::..  ** :** **: : **** *: .: .*  ::  ***:***  *:* :*:*

                             OB NTP BIND
MLE_DROME       PNICVHKEKRKVLTTESKAALLHKTSVNCSN-LAVTFPYPFFVFGEKIRTRAVSCKQLSM
MLE_ACYPI       PNVCYHKVKRKVYTTEGKFALIHKTSVNCNNTMDGSFQSPFFVFAEKVRTTAVSCKQMTM
                **:* ** **** ***.* **:*******.* :  :*  *****.**:** ******::*

MLE_DROME       VSPLQVILFGSRKIDLAANNIVRVDNWLNFDIEPELAAKIGALKPALEDLITVACDNPSD
MLE_ACYPI       VTPIHLLLFGARKIEY-TKELVQLDNWINLKMDVTAASAIVALRPAIESLIVHASEEPGS
                *:*::::***:***:  ::::*.:***:*:.::   *: * **.**:*.**. *.::*..

MLE_DROME       ILRLEEPYAQLVKVVKDLCVKSAGDFGLQRESGILPHQSRQFSDGGGPPKRGRFETGRFT
MLE_ACYPI       IAMLSETDIKLINVLKELCNFNCGRYNLSPIT---------FVQGDRPNYRRPF------
                *  *.*.  :*::*:*:**  ..* :.*.  :         * :*. *  *  *

MLE_DROME       NSSFGRRGNGRTFGGGYGNNGGGYGNNGGGYGNIGGGYGNNAGGYGNNGGYGNNGGGYRN
MLE_ACYPI       NQTFNESGDAS------DNKVQHINHSHSSYNSFQRG---GRGRYNEGIGYRGNSSSLCD
                *.:*.  *:.       .*:    .:. ..*..:  *   . * *.:. ** .*...  :

MLE_DROME       NGGGYGNNGGGYGNKRGGFGDSFESNRGSGGGFRNGDQGGRWGNF
MLE_ACYPI       EYNQRTYHDGGRSVREQGF-----TGYRSENGFKRC--------F
                : .    :.** . .  **     :.  * .**..         *
```
