# Supplementary material for: Dosage compensation and sex-specific epigenetic landscape of the X chromosome in the pea aphid
Source: Epigenetics Chromatin. 2017 Jun 15;10:30. doi: 10.1186/s13072-017-0137-1 (PMC5471693; doi:10.1186/s13072-017-0137-1)
Supplement: Supplementary file 7 — Additional file 7. Protein conservation of the Drosophila’s DCC in the pea aphid. Proteic domains percentage of identity between A. pisum homologs of the five proteins composing the DCC of D. melanogaster. [file 13072_2017_137_MOESM7_ESM.pdf]

Additional file 7: Protein conservation of the Drosophila's DCC in the pea aphid. Proteic domains percentage of identity between *A. pisum* homologs of the five proteins composing the DCC in *D. melanogaster*.

***D. melanogaster* protein name**	***A. pisum* homologs**	**Proteic domains**	***A. pisum and D. melanogaster* domain identity**
MSL1_DROME	XM_003243415.1	PEHE	0.28
MSL2_DROME	ACYPI49193	zf-RING10	0.25
		MSL2-CXC	0.32
MSL3_DROME	ACYPI000966	MRG	0.22
MOF_DROME	ACYPI002102	MOZ-SAS	0.67
MLE_DROME	ACYPI003650	dsrm	0.45
		DEAD	0.68
		Helicase_C	0.69
		HA2	0.43
		OB NTP BIND	0.57
